# Structural Insights and Reaction Profile of a New Unspecific Peroxygenase from *Marasmius wettsteinii* Produced in a Tandem-Yeast Expression System

**DOI:** 10.1021/acschembio.4c00504

**Published:** 2024-10-05

**Authors:** Israel Sánchez-Moreno, Angela Fernandez-Garcia, Ivan Mateljak, Patricia Gomez de Santos, Martin Hofrichter, Harald Kellner, Julia Sanz-Aparicio, Miguel Alcalde

**Affiliations:** † Department of Biocatalysis, Institute of Catalysis, CSIC, 28049 Madrid, Spain; ‡ Department of Crystallography & Structural Biology, Institute of Physical Chemistry “Blas Cabrera”, CSIC, 28006 Madrid, Spain; § EvoEnzyme S.L., C/Faraday 7, Parque Científico de Madrid, 28049 Madrid, Spain; ∥ Department of Bio- and Environmental Sciences TU Dresden, International Institute Zittau, Markt 23, 02763 Zittau, Germany

## Abstract

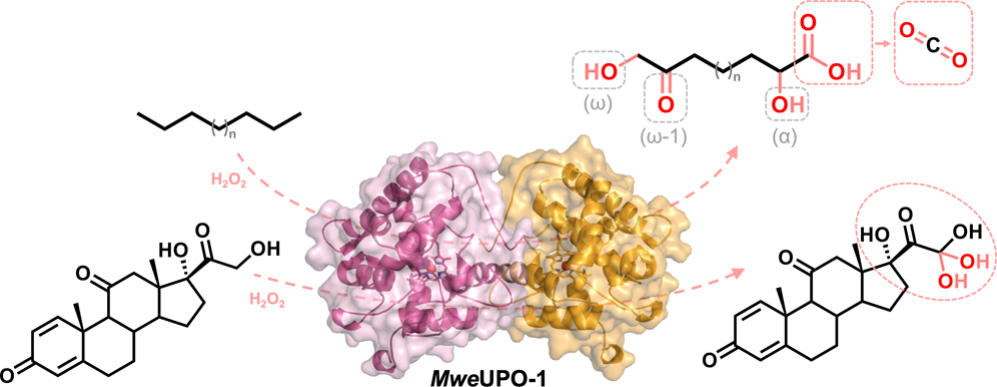

Fungal unspecific peroxygenases (UPOs) are gaining momentum in synthetic chemistry. Of special interest is the UPO from *Marasmius rotula* (*Mro*UPO), which shows an exclusive repertoire of oxyfunctionalizations, including the terminal hydroxylation of alkanes, the α-oxidation of fatty acids and the C–C cleavage of corticosteroids. However, the lack of heterologous expression systems to perform directed evolution has impeded its engineering for practical applications. Here, we introduce a close ortholog of *Mro*UPO, a UPO gene from *Marasmius wettsteinii* (*Mwe*UPO-1), that has a similar reaction profile to *Mro*UPO and for which we have set up a directed evolution platform based on tandem-yeast expression. Recombinant *Mwe*UPO-1 was produced at high titers in the bioreactor (0.7 g/L) and characterized at the biochemical and atomic levels. The conjunction of soaking crystallographic experiments at a resolution up to 1.6 Å together with the analysis of reaction patterns sheds light on the substrate preferences of this promiscuous biocatalyst.

## 1 Introduction

Over the past two decades, fungal unspecific peroxygenase (UPO, EC 1.11.2.1) has emerged as a versatile biocatalyst capable of performing selective C–H sp^3^ and C=C sp^2^ oxyfunctionalization reactions.^1^ As a member of the heme-thiolate-containing peroxidase superfamily, UPO activity is fueled simply by H_2_O_2_, which plays a dual role of oxygen donor and final electron acceptor. Upon heterolytic cleavage of H_2_O_2_ by a catalytic acid–base pair located in the neighborhood of the prosthetic heme group, the enzyme switches from its resting state via Compound 0 to the first catalytic intermediate Compound I, an oxo-ferryl cation π-radical complex of heme ([heme]^•+^-Fe = O) that underlies the strong reactivity of this versatile enzyme. As such, through a peroxygenative route (stepwise two-electron oxidation), UPO can carry out aliphatic, aromatic and heterocyclic hydroxylations/oxygenations, aliphatic and aromatic alkene epoxidations, *O*- and *N*-dealkylations (via unstable hemiacetal and hemiaminal intermediates), sulfoxidations and halogenations.^2,3^ Moreover, this enzyme catalyzes the one-electron (peroxidative) oxidation of diverse phenolic compounds. When compared to the classic cytochrome P450 monooxygenase, UPO is a simpler yet much more efficient and stable oxyfunctionalization biocatalyst, not least as it neither relies on complex redox cofactor nor auxiliary flavoproteins, and more importantly, it is not susceptible to oxidative uncoupling while achieving similar chemistries.^4−6^

With over 4000 putative UPO sequences deposited in genomic databases and with a widespread distribution within the fungal kingdom, UPOs have been classified phylogenetically into two different families with distinct structural properties and substrate scopes: short and long UPOs (also referred to as family I and family II, respectively).^3,7^ In general terms, long UPOs are monomeric proteins with a molecular weight of ∼44 kDa and with a strong preference toward small aromatic compounds due to their narrower and “carafe-shaped” heme channel (e.g., *Aae*UPO vide infra). By contrast, short UPOs are either dimeric or monomeric proteins with a molecular size of ∼26 kDa per monomer, harboring a wide open and symmetric cone-shaped channel (frustum) that dictates their preference for bulky substrates like steroids and long aliphatic compounds (a preference attributed to *Mro*UPO, see below).^8^

The UPO from *Agrocybe (Cyclocybe) aegerita* (*Aae*UPO), the first UPO described, is a canonical representative of family II. *Aae*UPO was the first of its class heterologously expressed in yeast in a functional/secreted form by means of directed evolution.^9^ The outcome of this lab evolution campaign was the evolved PaDa-I mutant, a highly active and stable UPO that is fully compatible with a tandem-yeast expression system, in which directed evolution experiments can be performed in *Saccharomyces cerevisiae*, producing these variants at high levels in *Pichia pastoris* (*Komagataella phaffii*).^10^*S. cerevisiae* is the host of choice for the directed evolution of complex fungal enzymes due to its versatility in terms of mutant library creation and screening: ease of manipulation, high transformation efficiencies, a broad variety of episomal vectors, and signal peptides driving secretion. Conversely, *P. pastoris* can reach cell densities that very few hosts can achieve in fed-batch bioreactors. Hence, this tandem-yeast expression system has been leveraged in recent years to undertake directed evolution campaigns with UPOs from different species, specifically targeting applications like bulk and fine chemical synthesis or the production of pharmaceuticals and their oxyfunctionalized metabolites (refs (11 and 12) and references therein).

**Scheme 1:**
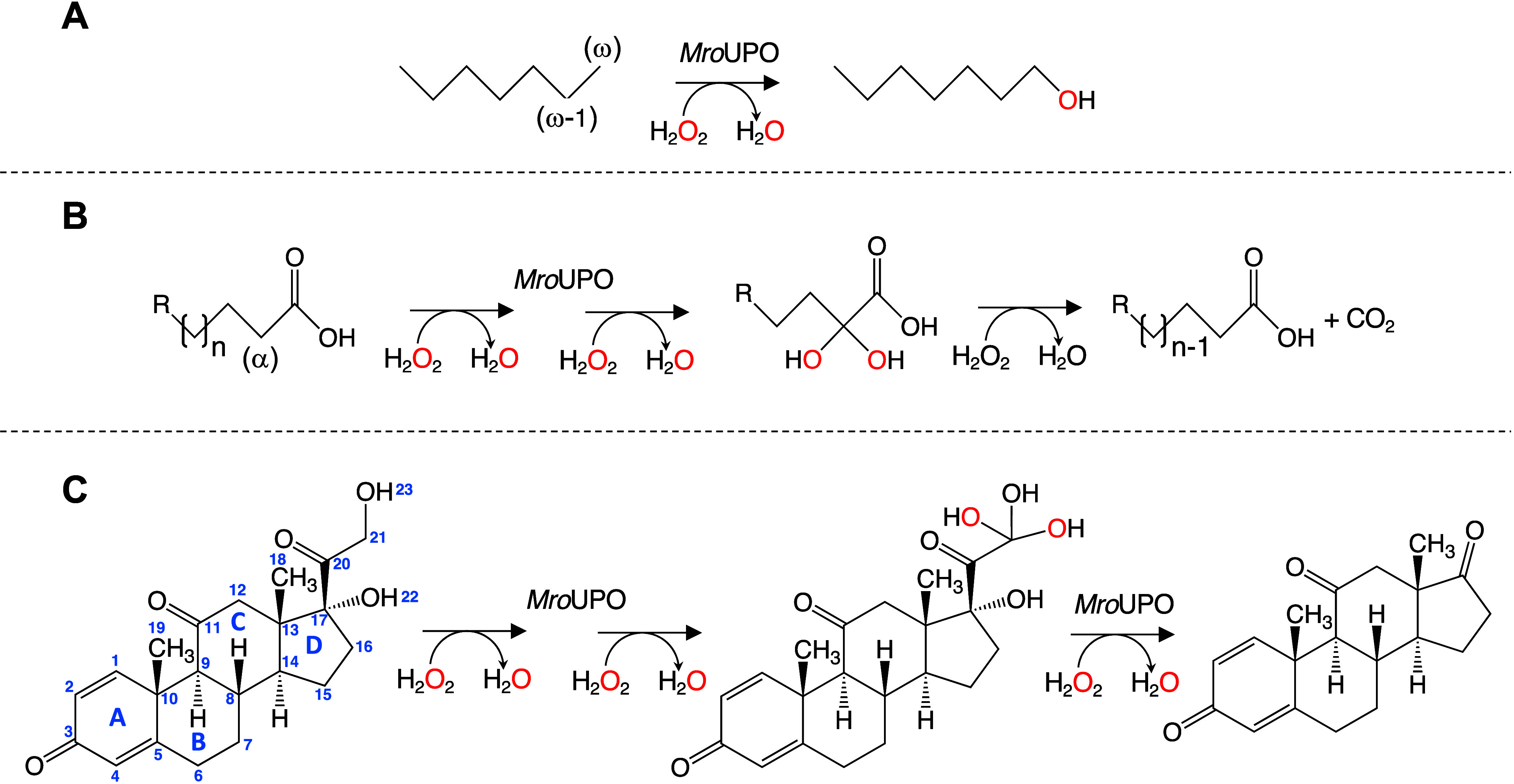
Reactivity of *Mro*UPO (A) Hydroxylation of *n*-alkanes at the terminal (ω) position. Depending on selectivity, further overoxidation reactions may lead to the formation of diols and (di)-carboxylic acids as well as ω-1 hydroxy- and keto-byproducts.^14^ (B) Fatty acid shortening through two stepwise (α) peroxygenation reactions, generating the intermediate *gem*-diol (ketone hydrate). The final decarboxylation of ketone by dehydration -in equilibrium with the *gem*-diol- is simply mediated by a hydroperoxide intermediate driven by H_2_O_2_.^15^ (C) Deacylation of cortiscosteroids (e.g., prednisone). Two stepwise peroxygenation reactions give rise to a *gem*-diol in equilibrium with the corresponding free aldehyde. Final side chain removal may occur by *Mro*UPO lyase-like activity or in a chemical reaction mediated by H_2_O_2_.^8^

On the other hand, the first reported short UPO was that from *Marasmius rotula* (*Mro*UPO), a homodimeric protein with an ample repertoire of selective oxygenation reactions, including the terminal hydroxylation of *n*-alkanes, the α-oxidation of fatty acids (fatty acid chain shortening-odd/even effect) and side-chain removal of corticosteroids (Scheme 1).^8,13−15^ Unfortunately, unlocking the huge potential of this *Mro*UPO in synthetic chemistry has been hindered by its poor functional expression in *S. cerevisiae*, limiting the possibility of performing practical engineering by directed evolution. Indeed, when we tried to express *Mro*UPO in *S. cerevisiae* and test different signal peptides in a modular yeast system,^16^ secretion was too weak to support a reliable directed evolution campaign (unpublished results). The structural organization of *Mro*UPO is complex, with a need to establish an intermolecular disulfide bridge between the monomers, and its particular dimeric organization apparently represents a bottleneck for productive heterologous expression. The few examples of engineering *Mro*UPO by rational design also highlight its limited expression in *Escherichia coli*, which is incompatible with the demanding high-throughput fermentation conditions of a directed evolution workflow (in terms of both culture growth and expression in 96-well plates).^17,18^

Here we present a new dimeric short UPO that can be heterologously expressed at high levels in both *S. cerevisiae* and *P. pastoris*, an enzyme that shares its substrate profile, selectivity, and biochemical and overall structural characteristics with *Mro*UPO. From the 58 putative short dimeric UPOs obtained from the phylogenetically related *Marasmius wettsteinii* genome (*Mwe*UPO), we identified a UPO gene that is 96% identical to that of *Mro*UPO in terms of protein sequence. Only 11 substitutions appeared to be responsible for its successful functional heterologous expression in *S. cerevisiae* and *P. pastoris* (on a g/L scale in a fed-batch bioreactor), two in the signal peptide, and nine in the mature protein. The recombinant *Mwe*UPO was characterized at the biochemical and atomic level, carrying out a thorough crystallographic and reaction study with a panel of representative substrates. These findings establish new possibilities for the engineering of *Mwe*UPO that are aimed at specific practical applications.

## 2 Results and Discussion

### 2.1 *Mwe*UPO Genome Mining, Heterologous Functional Expression in Yeast, and Production in a Bioreactor

The agarics *M. rotula* and *M. wettsteinii* are pinwheel mushrooms that differ primarily in terms of their preferred growth substrate (leaf litter vs needle litter). Thus, they are closely related species (“phylogenetic sisters”) such that their UPO sequences are likely to be very similar, differing at only a few amino acids. In 2018, we produced *Mwe*UPO homologously, and we demonstrated it had an identical reaction pattern to *Mro*UPO regarding the oxidation of steroids with hydroxyacetyl and hydroxyl functionalities at C21.^8^ In light of this, we mined the genome of *M. wettsteinii* in search of potential UPO sequences with similar substrate profiles to *Mro*UPO. From a set of 58 putative *Mwe*UPOs, we selected those with the highest sequence similarity to that of *Mro*UPO and tested their functional expression in *S. cerevisiae*. The gene g4356 located on contig0161 (from now on *Mwe*UPO-1) was successfully expressed in *S. cerevisiae* with surprisingly good titers (∼6 mg/L in 96-well plate fermentation, ∼12 mg/L in a flask (Supplementary Table S1)). The *Mwe*UPO-1 gene encodes a protein of 236 amino acids with a 28 amino acid signal peptide. This gene only differs from *Mro*UPO through 11 substitutions, two in the native signal peptide and the remaining nine in the mature protein (Supplementary Figure S1). These substitutions probably underlie the successful secretion by *S. cerevisiae*, a host in which *Mro*UPO is hardly expressed, while other UPO genes isolated from *M. wettsteinii* did not show peroxygenase activity when expressed in this host (Supplementary Figure S2).^16^ Indeed, we recently carried out ancestral sequence reconstruction (ASR) studies of the short UPO family, successfully expressing several ancestral nodes (unpublished results). Interestingly, when comparing *Mwe*UPO-1 with the ancestral nodes, we observed that four of the 9 substitutions of mature *Mwe*UPO-1 were actually ancestral mutations that may produce a beneficial effect on expressibility. While this result underlies the significance of ancestral mutations on the functional expression of short UPOs, we cannot rule out the potential beneficial effect of the rest of the *Mwe*UPO-1 substitutions on expression, something that can only be studied by performing a benchmark mutagenesis analysis, i.e. inserting such substitutions (individually or in combinations) onto the *Mro*UPO template.

All in all, *Mwe*UPO-1 surpassed the expression of the PaDa-I mutant by ∼1.5-fold, the latter being the result of five generations of directed evolution for functional expression in *S. cerevisiae*.^9^ In view of these results, we set up a tandem yeast expression system to perform laboratory evolution campaigns in *S. cerevisiae* and to overproduce *Mwe*UPO-1 variants in *P. pastoris* in a fed-batch bioreactor.^10^ This first objective was rapidly accomplished, achieving 5.7 mg/L *Mwe*UPO-1 secretion by *S. cerevisiae* in a microtiter plate with a variance coefficient of ∼12%, suitable to construct and explore mutant libraries in directed evolution experiments. We cloned the enzyme in *P. pastoris*, employing a strain screening protocol based on increasing amounts of the antibiotic zeocin that allowed optimal multicopy variants to be isolated and used for fermentation in a 5 L fed-batch bioreactor. Superb levels of production were achieved (690 mg/L) without optimizing the fermentation conditions, in the range of the UPOs most strongly expressed by *P. pastoris* to date, even including the long UPO from *Galerina marginata* and the short UPO from *Aspergillus brasiliensis*.^19,20^

### 2.2 Biochemical Characterization

Recombinant *Mwe*UPO-1 secreted by *P. pastoris* was purified to homogeneity (Reinheitszahl value [*Rz*] *A*_418_/*A*_280_ ∼ 2) and its main biochemical properties were assessed: thermostability, pH activity optima for three substrates, as well as kinetic and spectroscopic properties (Table 1 and Supplementary Figure S3).

**Table 1:** Biochemical Features of the Recombinant *Mwe*UPO-1 and *Mro*UPO

biochemical feature	*Mwe*UPO-1^a^	*Mro*UPO^b^
mass (Da)^c^	35,000	32,000
mass (Da)^d^	35,090	n.d.
mass (Da)^e^	25,540	25,650
degree of glycosylation (%)^f^	27	20
thermal stability (*T*_50_, °C)^f^	54.4	n.d.^h^
pI	5.5	5.27
optimum pH for ABTS^i^	4.0	4.5
optimum pH for DMP^j^	5.0	5.5
optimum pH for VA^k^	5.0	5.5
*Rz* (*A*_418_/*A*_280_)	2.0	n.d.^h^
Soret region (nm)	418	418
CT1 (nm)^g^	570	570
CT2 (nm)^g^	536	536

a Recombinant *Mwe*UPO-1 expressed in *P. pastoris*.

b *Mro*UPO wild-type from the original fungus (data from ref (13)).

c Estimated by SDS-PAGE.

d Estimated by MALDI-TOF mass spectrometry.

e Estimated from the amino acid composition (ProtParam).

f Estimated from the purified enzyme.

g CT1 and CT2: charge transference bands 1 and 2, respectively.

h n.d.: not determined.

i ABTS (2,2′-azino-bis(3-ethylbenzothiazoline-6-sulfonicacid)).

j DMP (2,6-dimethoxyphenol).

k VA (veratryl alcohol).

**Figure 1:**
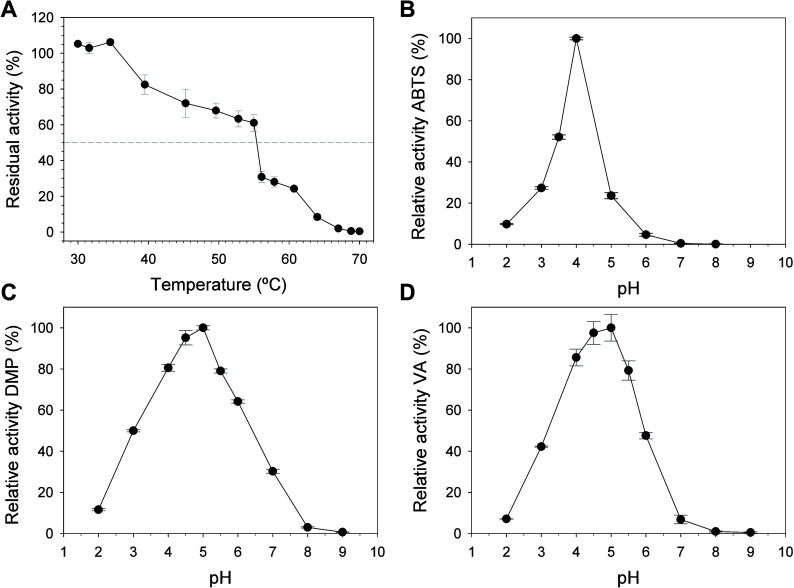
Biochemical characterization of *Mwe*UPO-1 expressed by *P. pastoris*. (A) Thermostability (*T*_50_). (B–D) pH activity profiles: these activities were measured in 100 mM Britton–Robinson buffer at different pH values with 2 mM H_2_O_2_ and 1 mM ABTS (B) and VA (D), or with 2 mM H_2_O_2_ and 2 mM DMP (C). UPO activity was normalized to the optimum activity value, and each point represents the mean and standard deviation of 3 independent experiments.

The average molecular mass estimated by MALDI-TOF-MS was 35 090 Da, with 27% glycosylation (Table 1 and Supplementary Figure S3). The spectroscopic profile of the enzyme was typical of a fungal peroxygenase, with a maximum in the Soret region around 418 nm and 2 Q-bands at 570 and 536 nm (Supplementary Figure S3). Kinetic thermostability was assessed through *T*_50_ (the temperature at which the purified enzyme retained 50% of its activity after a 10 min incubation), obtaining *T*_50_ values of 54.4 °C that lie within the range of other recombinant and stable UPOs expressed by yeast^10,21^ (Figure 1A and Table 1).

The pH profiles for peroxidative (with ABTS, DMP) and peroxygenase (with VA) activities revealed similar trends and optimum pH values (4.0, 5.0, and 5.0 for ABTS, DMP, and VA, respectively) (Figure 1B–D and Table 1).

Kinetic constants for peroxidative and peroxygenative activities were assayed, confirming the similar catalytic activity between the recombinant *Mwe*UPO-1 and wild-type *Mro*UPO (Table 2).

**Table 2:** Kinetic Parameters of Recombinant *Mwe*UPO-1 and Wild-Type *Mro*UPO

	*Mwe*UPO-1^a^			*Mro*UPO^b^		
	*k*_cat_ (s^–1^)	*K*_m_ (mM)	*k*_cat_/*K*_m_ (M^–1^ s^–1^)	*k*_cat_ (s^–1^)	*K*_m_ (mM)	*k*_cat_/*K*_m_ (M^–1^ s^–1^)
VA	22.46 ± 0.73	0.26 ± 0.08	8.76 × 10^4^ ± 27,143	49	0.279	1.76 × 10^5^
ABTS	15.49 ± 0.68	0.03 ± 0.003	5.34 × 10^5^ ± 6009	25	0.071	3.53 × 10^5^
DMP	61.57 ± 0.85	0.22 ± 0.001	2.85 × 10^5^ ± 4228	70	0.133	5.29 × 10^5^
H_2_O_2_	60.92 ± 0.83	1.39 ± 0.292	4.40 × 10^4^ ± 9266	76	3.14	2.42 × 10^4^

a Recombinant *Mwe*UPO-1 expressed in *P. pastoris*.

b Wild-type *Mro*UPO from the original fungus (data from ref (13)).

Kinetic parameters were calculated for each active site. ABTS kinetic constants for *Mwe*UPO-1 were estimated in 100 mM Britton–Robinson buffer pH 4.0, containing 2 mM H_2_O_2_; and those for DMP and VA in 100 mM potassium phosphate buffer pH 5.0, containing 2 mM H_2_O_2_. The H_2_O_2_ kinetic constants were estimated using veratryl alcohol as a reducing substrate under the corresponding saturated conditions.

## 3 Crystallography Study and Analysis of the Reaction Patterns

### 3.1 *Mwe*UPO-1 Crystal Structure: General Features

When expressed in *P. pastoris*, *Mwe*UPO-1 was heavily glycosylated. To reduce sample heterogeneity that can obstruct crystal growth, the enzyme was deglycosylated by treatment with Endo (endoglycosidase) H. The activity of *Mwe*UPO-1, in both glycosylated and deglycosylated, forms was similar, which is in good agreement with our former observations for the PaDa-I mutant before and after deglycosylation.^22^ High-quality large plate crystals were grown in 10–20% (v/v) PEG 4000/0.2 M (NH_4_)_2_SO_4_, and the crystals diffracted to a maximum resolution of 1.58 Å. The crystals belong to the *P*4_3_2_1_2 space group, with two molecules per asymmetric unit (Supplementary Table S2). The electron density maps allowed the whole polypeptide chain to be modeled, from Ser1 to Leu236. The model contains the characteristic heme-thiolate prosthetic domain, the structural Mg^2+^ ion, and any remaining GlcNAc units attached to the Asn35, Asn122, and Asn143 N-glycosylation sites. In addition, several glycerol molecules from the cryoprotectant solution were bound at different positions on the molecule’s surface.

**Figure 2:**
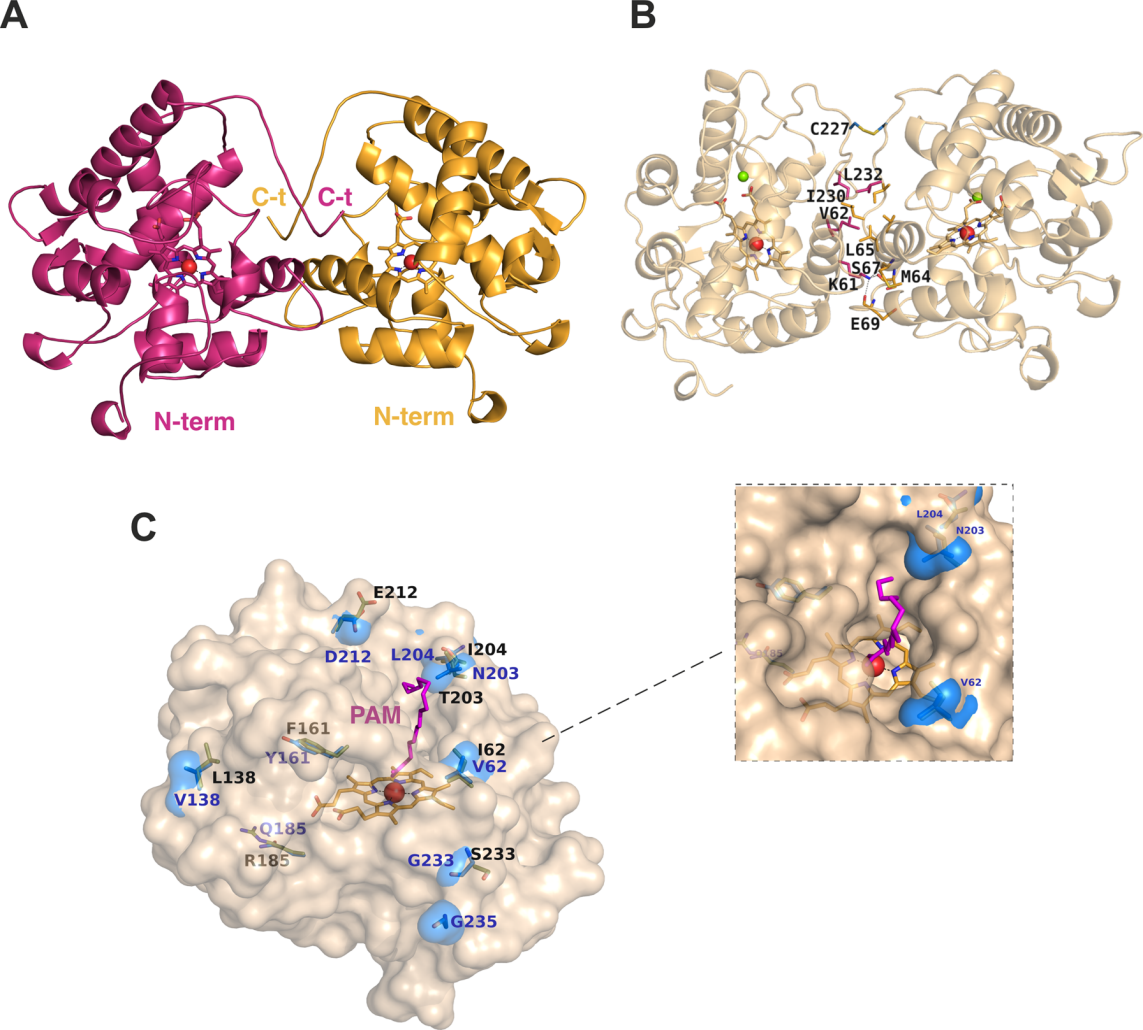
Structural overview of *Mwe*UPO-1. (A) Ribbon diagram displaying the two subunits of the homodimer in orange and pink. The C-terminal domain of one subunit is on top and forms the heme access channel with the other. (B) Dimer interface of *Mwe*UPO-1. The heme and amino acid chains involved in the contacts between the subunits are depicted as sticks. The intermolecular disulfide bridge mediated by Cys227 is shown through orange sticks, while polar contacts are shown as dashed lines. (C) Surface representation of the *Mwe*UPO-1 subunit showing in blue sticks sequence differences with respect to *Mro*UPO (PDB entry 5FUK) that is represented in yellow sticks. A bound palmitic acid (PAM) captured in the *Mro*UPO crystal is represented to highlight the heme channel. The inset underlines the Val62 position within the *Mwe*UPO-1 heme channel and Leu204 protruding at its entrance.

The asymmetric unit of the *Mwe*UPO-1 crystal structure consists of two identical polypeptide chains that are arranged in a homodimer (Figure 2A). The monomer is mostly helical, and it is comprised of 11 α-helices and two short β-strands. These monomers are covalently linked through an intermolecular disulfide bridge between Cys227 of both polypeptides, and many hydrophobic contacts are formed among the Val62, Leu65, Ile230, and Leu232 residues in each chain. In addition, the Lys61 side-chain establishes polar contacts with the Met64, Ser67, and Glu69 backbone carbonyls from the adjacent subunit (Figure 2B). Apart from Val62, which is an Ile62 residue in *Mro*UPO (PDB entry 5FUK), these residues are all conserved between both enzymes, producing an equivalent dimeric interface. The mature *Mwe*UPO-1 protein has 96% sequence identity with *Mro*UPO, with a series of substitutions distributed along the polypeptide chain (Figure 2C): *Mro*UPO/*Mwe*UPO-1-Ile62Val, Leu138Val, Phe161Tyr, Arg185Gln, Thr203Asn, Ile204Leu, Glu212Asp, Ser233Gly, and Glu235Gly. Among these, the *Mwe*UPO-1 residues Val62 and Leu204 are located near (Leu204) or at the heme channel (Val62) somehow shaping a wider entrance to the tunnel and, hence, potentially influencing the binding of bulky ligands as will be shown below. Interestingly, subunit cooperativity is observed between the two polypeptide chains since the C-terminal Leu236 of one subunit lies on top and forms the heme access channel of the other (Figure 2A). Although far from the heme group, both C-terminal regions are involved in substrate binding (vide infra).

**Figure 3:**
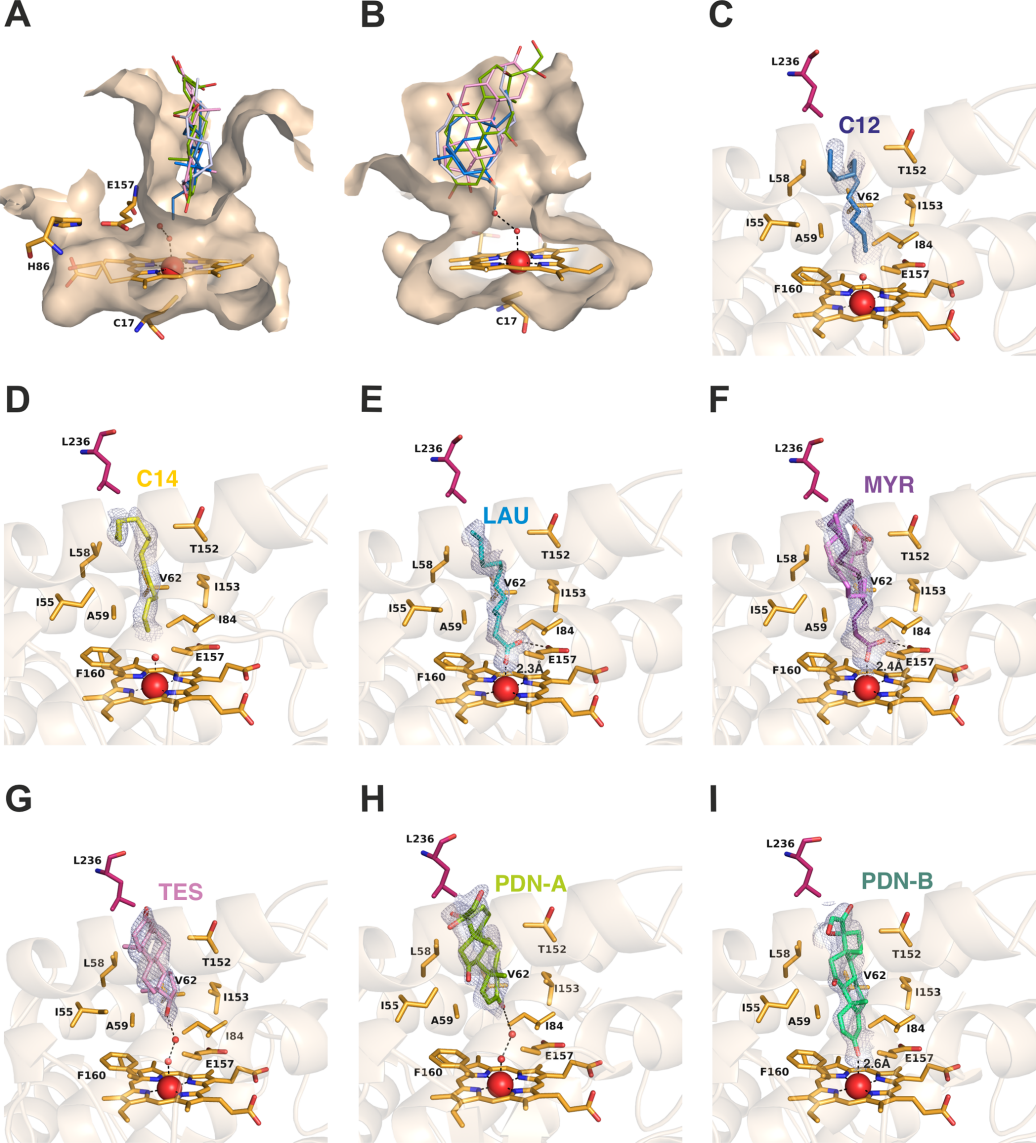
Crystallographic structure of the *Mwe*UPO-1 complexes. (A and B) Two views of the *Mwe*UPO-1 heme channel showing the ligands bound to the different crystal complexes, which have been structurally superimposed to the holo structure. Water molecules at the apoenzyme are included as red spheres. Complexes with (C) dodecane (C12), (D) tetradecane (C14), (E) lauric acid (LAU), (F) myristic acid (MYR) found in two alternate conformations, (G) testosterone (TES), and (H, I) prednisone (PDN) bound to molecules A and B. The relevant residues defining the heme channel are represented as sticks (in orange) and polar bonds are shown as dashed lines, while the Leu236 from the adjacent subunit is represented by pink sticks. Water molecules involved in substrate recognition are included as red spheres. See also Supplementary Figures S5 and S6 for the complexes with isophorone and limonene.

The heme protoporphyrin IX (heme group) is at the bottom of a long channel, its iron being hexa-coordinated with its fifth position linked to the proximal Cys17 ligand and the sixth position (distal ligand) occupied by a water molecule (Figure 3A, B; see the general catalytic mechanism in Supplementary Figure S4). Interestingly, there is a clear density where a second water molecule establishes a polar link to this distal water molecule, putatively mimicking the early ferric-peroxo complex (“pre-compound 0”) formed in the first step of the postulated catalytic cycle of UPOs. The heme access cavity is a 17 Å long, deep funnel with a wide irregularly shaped entrance that reduces to a narrow base close to the heme group. This constricted base is defined by the residues Ala59, Ile84, Glu157, and Phe160 (Figure 3C). From these, the acidic residue forms the Glu157–His86 catalytic acid–base pair (Figure 3A) required for binding and heterolytic cleavage of peroxide, while Ile84 and Phe160 (Figure 3C) are equivalent to the Phe121 and Phe199 residues in PaDa-I, both included in the characteristic aromatic tripod that directs substrate binding to the heme.^22^ In *Mwe*UPO-1, the equivalent position to the third aromatic residue (Phe69 in PaDa-I) would be Val51, far from the heme, while Ala59 is involved in substrate recognition, as will be shown below. On the other hand, the funnel is shaped by many hydrophobic, nonaromatic residues, mostly leucine and isoleucine, with only the polar Thr152 and Ser156 being distal to the iron.

### 3.2 Soaking Experiments and Analysis of the Enzymatic Reactions

*Mwe*UPO-1 crystals were studied by soaking experiments with a panel of the characteristic substrates, all of which were analyzed during the corresponding enzymatic reactions. We assayed alkanes (dodecane and tetradecane), monocarboxylic fatty acids (lauric acid and myristic acid), steroids (testosterone and prednisone), and terpenoids (*R*-limonene and isophorone) (Figures 3, S5, and S6). Full details about the crystallography of *Mwe*UPO-1 complexes can be found in the Materials and Methods section and in Supplementary Table S2.

The clear electron density obtained in all the complexes illustrates ligand binding within the heme channel, a phenomenon for which evidence had already been found in previous spectroscopic binding studies with wild-type *Aae*UPO.^23^ However, only the long flexible chains of the alkanes and the fatty acids could be accommodated at catalytically relevant positions near the iron or the oxo ion, while the bulky ligands were apparently positioned too far from the heme group to assist in catalysis. This distant prepositioning of a substrate was observed previously in the deposited complex of *Mro*UPO with racemic propranolol (PDB code 5FUK), although in that case propranolol partially occupied the active site and an alternate palmitic acid moiety was also observed.

Nevertheless, these observations suggest a general trend in the binding of bulky substrates to short UPOs. The rationale for this precatalytic binding mode might reside in the geometric constrictions imposed by the narrow heme channel base (as described above), which may prevent the crystallographic capture of less energetically favored binding positions that might represent productive complexes. Another interesting feature of the *Mwe*UPO-1 heme channel is the absolute lack of ordered water molecules, even in the ligand-free crystals. This situation contrasts with the PaDa-I crystals where the funnel is occupied by an extended net of water molecules, ordered through hydrogen bonds with the main chain of different residues that are displaced upon substrate binding.^22^ This supports the extraordinarily strong hydrophobicity of the *Mwe*UPO-1 heme funnel.

#### 3.2.1 Alkanes and Fatty Acids

Long-chain substrates, alkanes (dodecane and tetradecane), and fatty acids (myristic acid and lauric acid) have a conserved “buried” mode of binding in close proximity to the heme (Figures 3C–F and S5A). This type of binding maximizes the hydrophobic interactions between the main part of their corresponding chains to multiple residues establishing the contour of the heme channel (Ile55, Leu58, Val62, Thr152, Ile153). It seems apparent that these hydrophobic interactions govern the binding mode shared by all of these ligands, as the two alkanes fail to establish a polar link to *Mwe*UPO-1 residue Glu157 made by the carboxylate of the fatty acids. This mode of binding makes it clear that only the terminal C1/C2 positions can effectively approach the Compound I catalytic species (oxo-ferryl iron) for hydroxylation, which might explain the ability to perform terminal hydroxylations on linear alkanes previously only reported for the closest *Mwe*UPO-1 homologue, *Mro*UPO.^14^

**Figure 4:**
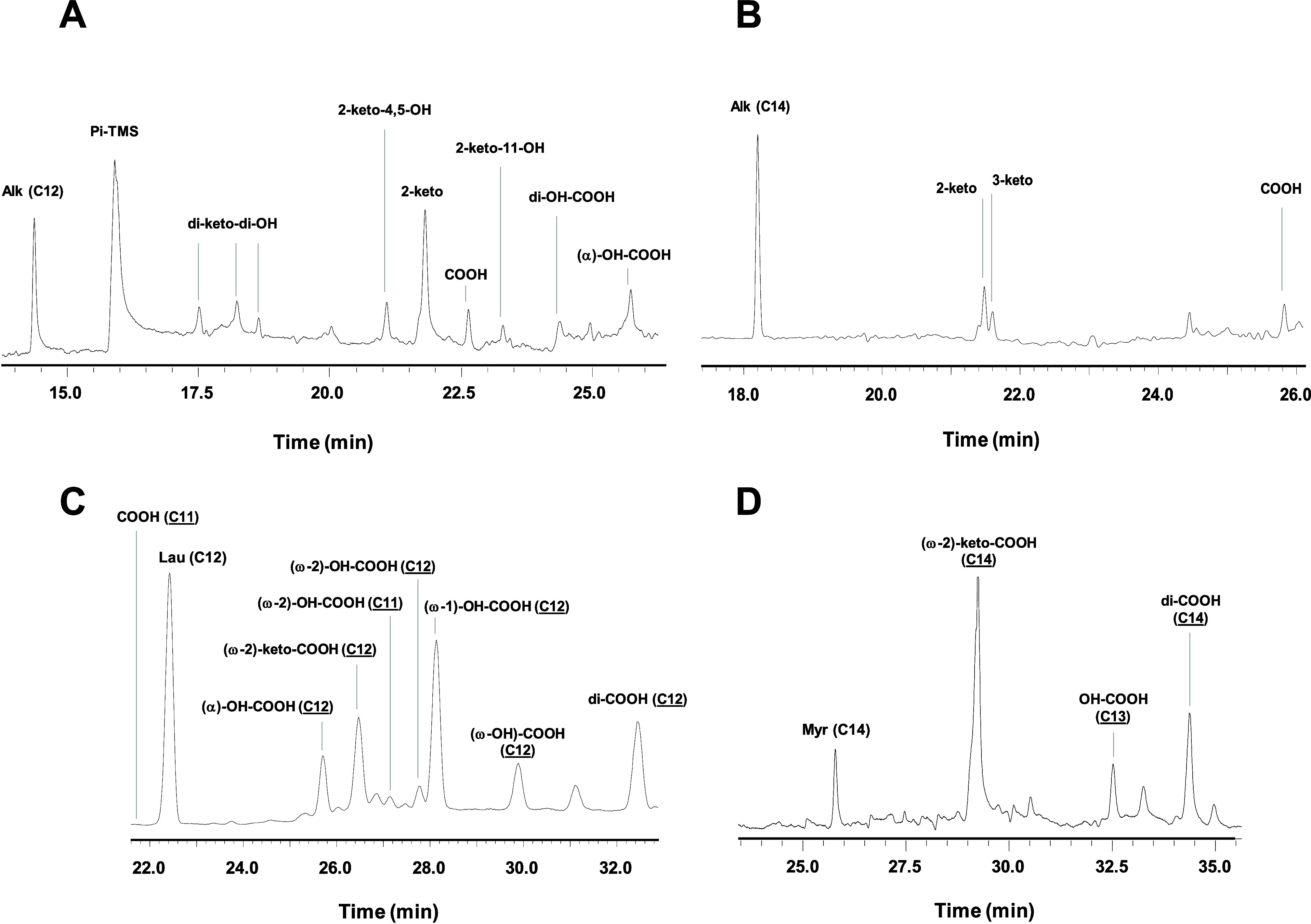
GC/MS analysis of the *Mwe*UPO-1 reaction with dodecane (A), tetradecane (B), lauric acid (C), and myristic acid (D). The reactions were performed in 50 mM potassium phosphate buffer pH 5.5, containing 0.3–0.5 mM of the substrate, 2 mM H_2_O_2_ and acetone 20% (v/v), and they were incubated for 1.5 h at 30–40 °C. The formation of carboxylic (COOH), dicarboxylic acids (di-COOH) ketones, terminal hydroxylation, and fatty-acid shortening was detected (mass spectra of reaction products are included in the Supporting Information).

To check the selectivity of *Mwe*UPO-1, enzymatic reactions were performed with dodecane, tetradecane, lauric acid, and myristic acid as substrates, and its hydroxylating activity was confirmed by gas chromatography–mass spectrometry (GC/MS (Figure 4). Significantly, the same oxidation pattern described for *Mro*UPO was observed for *Mwe*UPO-1, which was capable of performing a cascade of mono- and diterminal oxidation reactions. These reactions produced the corresponding fatty alcohols and the concomitant di/carboxylic acids via *gem*-diol intermediates (i.e., hydrated form of aldehyde), as well as inducing fatty acid shortening via terminal oxidative decarboxylation.^16,17^

Indeed, while most UPOs can hydroxylate fatty acids, mainly at the ω1/ω2 positions, the progressive one-carbon shortening of mono- and dicarboxylic acids is a feature exclusively observed for *Mro*UPO and *Mwe*UPO-1 (Scheme 1). As indicated above, a palmitic acid molecule was trapped in the *Mro*UPO crystals with its carboxylate coordinated to the iron (PDB 5FUK), illustrating this chain-shortening activity. Here, the captured lauric acid confirms this capacity, with its carboxylate coming into close contact with the heme and enabling shortening (Figure 3E). However, two alternative fatty acid binding positions were detected in the myristic complex, one resembling the lauric complex, while the second is a fully bent conformation that partially occupies the entrance to the channel (Figure 3F). This second binding mode might represent a preliminary state prior to the subsequent formation of a fully extended conformation that would situate the aliphatic chain closest to the activated oxo-heme group at terminal or subterminal positions for hydroxylation. As such, the results obtained by soaking *Mwe*UPO-1 crystals in lauric or myristic acid may be explained by the dependence on chain length described for monocarboxylic fatty acid shortening by *Mro*UPO.

#### 3.2.2 Steroids and Terpenoids

The ability of *Mro*UPO and two other fungal peroxygenases to catalyze the regioselective oxyfunctionalization of a representative series of steroids has previously been analyzed.^24^ Accordingly, hydroxylation at the side chain of ring D was preferred over that of the steroidal rings, although some hydroxylated derivatives at the gonane core were formed when oxygen groups were absent at C3. Consequently, testosterone, which lacks the alkyl moiety and is oxyfunctionalized at the C3 position was not a substrate of *Mro*UPO. In a later study, we described a new short-type, monomeric UPO from the ascomycetous mold *Chaetomium globosum* (*Cgl*UPO) that acts on testosterone, mostly through the selective epoxidation of the unsaturated alicyclic A ring of the gonane core, although hydroxylation of the D ring was also realized albeit to a much lesser extent.^25^ Here, we have trapped testosterone in the *Mwe*UPO-1 channel (Figures 3G and S5B,C), tightly packed between Ile55 and Leu58 at one face, and between Thr152 and Ile153 at the other, with its D ring facing the heme in a manner that apparently represents the less favored binding mode described for *Cgl*UPO. In this orientation, the hydroxyl group of cyclopentane makes a hydrogen bond with the pair of water molecules initially indicated in the ligand-free structure, putatively mimicking the ferric-(hydro)peroxo complex(es), representing the first catalytic intermediates pre-Compound 0 [heme-Fe^3+^-OOH_2_] or Compound 0 [heme-Fe^3+^-OOH]. This arrangement might subsequently bring closer the contiguous C16 position to become oxygenated and produce 16*a*-hydroxytestosterone. However, when we followed the enzymatic reaction of *Mwe*UPO-1 with testosterone by GC/MS and HPLC, we did not observe any conversion of this compound, consistent with the behavior of *Mro*UPO. While *Mwe*UPO-1 is unable to convert testosterone, our results show that the steroid core does diffuse through the heme channel, establishing chemically significant interactions with the catalytic site. These data shed light on a potential binding mode that may be amenable to catalysis upon structure-guided evolution.

**Figure 5:**
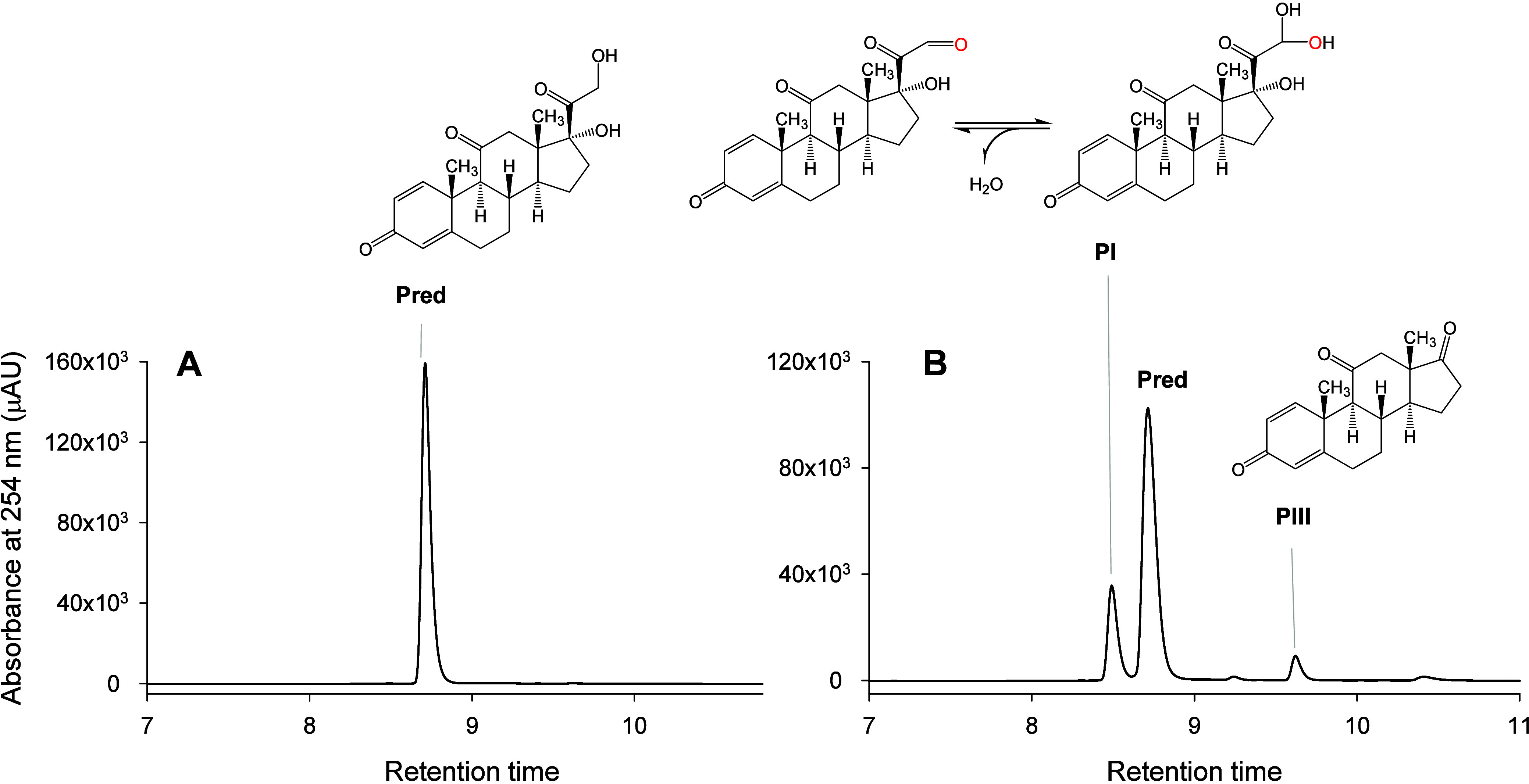
HPLC analysis of the *Mwe*UPO-1 reaction with prednisone (B), compared to the control reaction in the absence of the enzyme (A). Reactions were performed in 50 mM potassium phosphate buffer pH 5.5, containing 0.5 mM substrate, 2 mM H_2_O_2_ and acetone 5% (v/v), and they were incubated for 2 h at 30 °C. Pred: prednisone, PI: prednisone 21-*gem-*diol, PIII: delta-1-adrenosterone.

Soaking of *Mwe*UPO-1 crystals with the corticosteroid prednisone led to opposite results than soaking with testosterone, i.e., the prednisone bound presents its A ring pointing to the heme group with the (per)oxygenated C3 being closest to the iron. Noticeably, two different sites of the steroid were observed in molecules A and B within the asymmetric crystal unit. At one more distant position, the ketone group is linked through a pair of water molecules (PDN-A, Figures 3H and S5B), while a second position locates the ketone group coordinated with the iron (PDN-B, Figure 3I). Thus, the orientation of the steroid ring in both binding positions seems to contradict the side-chain oxidation activity of corticosteroids reported for *Mro*UPO and *Mwe*UPO. Indeed, this orientation implies proximity of the D-ring, with its side chain directed toward the reactive heme site, eventually leading to stepwise oxygenation and the final fission of the side chain.^8^ Consequently, the prednisone molecules trapped in our crystals might represent either a nonproductive complex (dead-end adduct) or possibly less favored binding modes that could lead to undetected secondary oxidation/(per) oxygenation products. Nevertheless, any artifact of the crystal soaking impeding the correct approach of prednisone to the heme leading to the productive complex cannot be discarded. To answer this question, we studied the reaction of *Mwe*UPO-1 with prednisone and the results were consistent with those described previously for the wild-type *Mro*UPO and *Mwe*UPO isoenzymes.^8^ The HPLC elution profile is consistent with the previously reported consecutive hydroxylation in the side chain of corticosteroids, which results in the formation of prednisone *gem*-diol in position 21 (PI) and delta-1-adrenosterone (PIII) by oxidative side chain removal (Figure 5). Similar distribution and retention times of the new products were reported with *Mwe*UPO under analogous reaction conditions and using comparable analytical methods.^8^ In addition, both the substrate (prednisone) and the products gave almost identical UV–visible spectra, indicating that the chromophore (double bonds of the steroidal A ring) in the oxidized molecules was not altered. Moreover, the formation of detectable amounts of a more hydrophobic product in longer reactions with *Mwe*UPO-1 suggested further oxidations of the already oxyfunctionalized prednisone, resulting in side chain removal due to decarboxylation of the carboxylic group to form delta-1-adrenosterone (PIII) (Figure 5).

**Figure 6:**
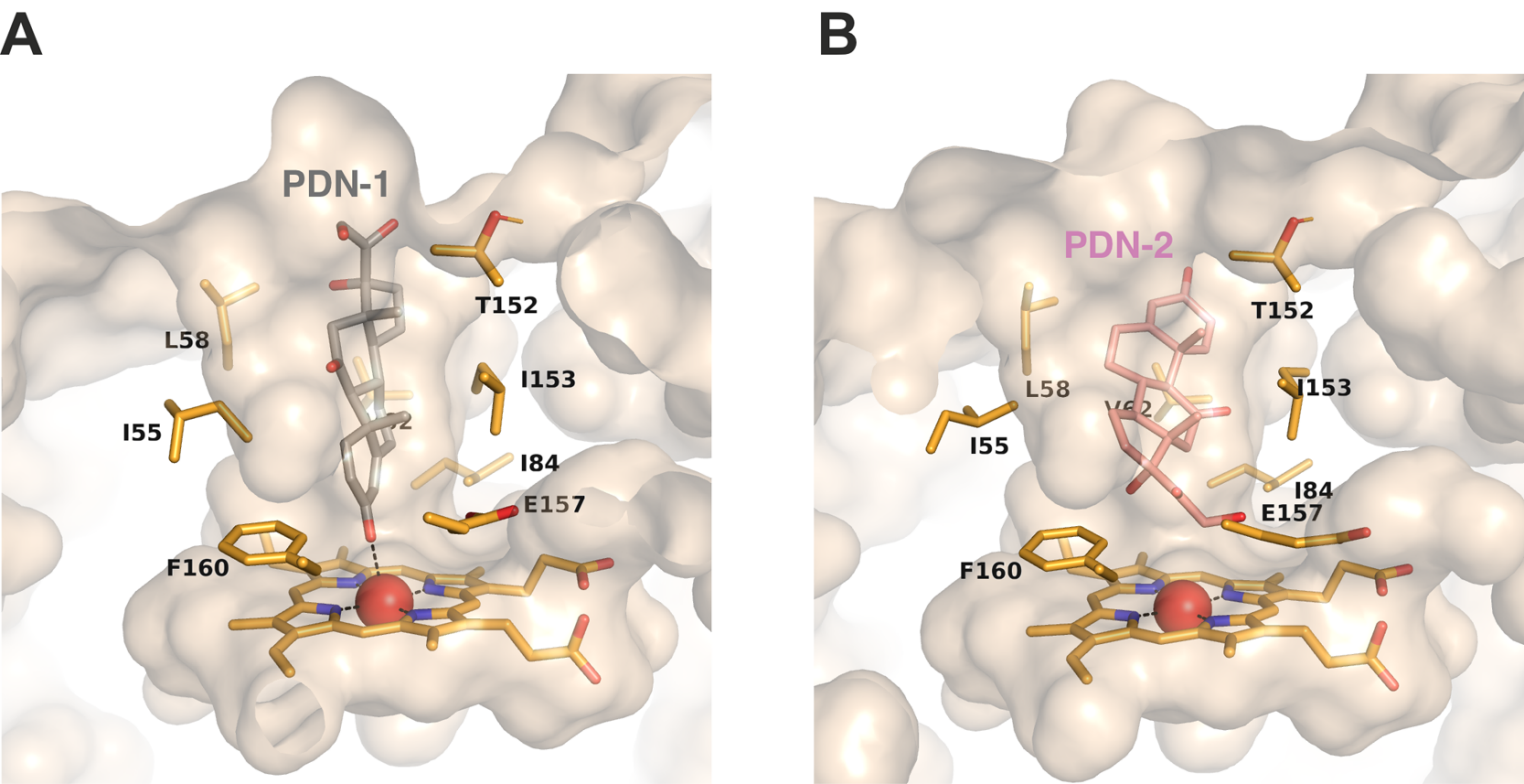
Automatic docking analysis of binding of prednisone (PDN) to the active site of *Mwe*UPO-1. The two poses with minimum binding energy are depicted in (A) and (B), with relevant residues contouring the heme channel and the heme group represented as sticks (in orange). The molecular surface of the channel is colored light brown.

In an attempt to understand the discrepancies between the crystal structure and the activity of recombinant *Mwe*UPO-1 on prednisone, we docked this substrate onto the active site of the enzyme. The automatic molecular docking data indicated two different binding modes displayed in 16 possible poses, and the two poses with the lowest binding energy (−10.1 and −9.2 kcal/mol) were selected. Interestingly, in the solution with minimal binding energy (Figure 6A), prednisone approached the heme, which is consistent with our crystals (PDN-B, Figure 3I). By contrast, the second binding mode inverts the position of prednisone, with the alkyl group facing the heme (Figure 6B), and correctly oriented to cause the removal of the side chain as observed in the analysis of the reaction (Figure 5). In this case, Glu157 is displaced, leaving room to allocate the alkyl moiety of the substrate. Therefore, it seems plausible that the most stable UPO-substrate complex has been trapped in the crystal, hence discarding a soaking artifact, and it was also modeled by automatic docking. The addition of the activating cosubstrate (H_2_O_2_) may result in a position change corresponding with the side-chain removing activity detected in both the reaction and the docking analysis.

Finally, soaking experiments performed with isophorone and limonene showed these molecules entering into the *Mwe*UPO-1 active site to establish tight hydrophobic interactions (Supplementary Figure S6), yet in less favored or unobserved binding modes when compared with other active UPOs that act on these compounds.^26^ This explains why we could not detect any activity on these substrates after analyzing the enzymatic reaction by GC/MS, which addresses the need to reshape the active site of *Mwe*UPO-1 to enable these compounds to be oxidized.

All in all, our results indicate a highly dynamic binding mode of compounds of different natures in the *Mwe*UPO-1 heme channel, which is in concordance with previous engineering studies computationally assisted by molecular dynamics and QM/MM simulations performed with both short and long UPOs.^27−31^

## 4 Conclusions

Advances in the field of UPO engineering are being demanded more and more by industry, in an age in which custom-made versions of these biocatalysts are beginning to be commercialized for different purposes and with many UPOs in the pipeline for heterologous expression and engineering.^11,12^ Among the wide diversity of UPOs, *Mro*UPO offers a unique portfolio of oxyfunctionalization reactions that include terminal hydroxylation of *n*-alkanes, fatty acid shortening, and side chain oxidation of corticosteroids. Despite this unique pattern of oxyfunctionalization reactions, its translation to industrial processes has hitherto been impeded by the lack of hosts that (over)express the enzyme and in which laboratory evolution experiments can be performed, and those that guarantee upscale production. The *Mro*UPO orthologue presented here, *Mwe*UPO-1, is easily adapted to a tandem yeast expression system for laboratory evolution and overproduction while completing the same reactions as *Mro*UPO. The characterization of the binding modes of different substrates, including some that should be not transformed by the enzyme, will help design structure-guided evolution in order to make *Mwe*UPO-1 a “dream biocatalyst” for the pharmaceutical and chemical industries. Finally, it is worth noting that several UPO crystal structures are now available, including the recent *art*UPO that shares ∼70% sequence identity with *Mwe*UPO-1, opening a venue for future protein chimeragenesis studies.^11,12,32^

## 5 Materials and Methods

### 5.1 Strains and Chemicals

Zeocin was purchased from Invitrogen (USA). The *Escherichia coli* strain XL1-Blue competent cells were obtained from Agilent Technologies (USA). The uracil-independent and ampicillin resistance shuttle vector pJRoC30 was obtained from the California Institute of Technology (CALTECH, USA). The protease-deficient *S. cerevisiae* strain BJ5465 was obtained from LGCPromochem (Barcelona, Spain). *Pichia pastoris* strain BG11 was purchased from Atum (USA). The plasmid used (pBSY5Z) was provided by Bisy (Austria). Restriction endonucleases *Not*I, *Bgl*II, *Pme*I, *Bam*HI, and *Xho*I together with Gibson Assembly Master Mix were purchased from New England Biolabs (USA). iProof High-Fidelity DNA Polymerase was purchased from Bio-Rad (USA). Oligonucleotide primers and UPO genes were acquired from Integrated DNA Technologies (USA). The NucleoSpin plasmid kit, NucleoSpin Gel, and PCR cleanup kit were purchased from Macherey Nagel (Germany). The zymoprep yeast plasmid miniprep kit was obtained from Zymo Research (Orange, CA, USA). ABTS (2,2′-azino-bis(3-ethylbenzothiazoline-6-sulfonicacid)) was purchased from Panreac AppliChem (Germany); DMP (2,6-dimethoxyphenol) was from TCI Europe (Switzerland). H_2_O_2_, (VA) veratryl alcohol, dodecane, tetradecane, lauric acid, myristic acid, testosterone, prednisone, *R*-limonene, and isophorone were purchased from Merck Life Science (USA). All chemicals and media components were of the highest purity available.

### 5.2 Mining *Marasmius wettsteinii* Genome

*Marasmius wettsteinii* was whole-genome sequenced using standard protocols with an Ion GeneStudio S5 System (Thermo Fisher Scientific) and subsequently assembled, their genes predicted and annotated.^33^ UPO genes were identified using the *M. rotula* reference (5FUJ_A) and a blastp search against the *M. wettsteinii* proteins. The closest match g4356 with 96.2% pairwise identity located on contig0161, designated as *Mwe*UPO-1, was used for further studies. Altogether, 58 short UPO genes were detected in *M. wettsteinii*. All data were submitted to the National Center for Biotechnology Information (NCBI). The raw data of the 49.1 Mpb genome were submitted to the Short Read Archive (SRA) under BioProject PRJNA930166 (BioSample number: SAMN40199879). The *Mwe*UPO-1 gene was deposited at GenBank under accession number PP399146.

### 5.3 Cloning of *Mwe*UPO-1 in *S. cerevisiae*

*Mwe*UPO-1 sequence was synthesized (with 50 bp overhangs to promote homologous recombination in *S. cerevisiae*) and cloned under the control of the GAL1 promoter of the pJRoC30 expression shuttle vector. *Bam*HI and *Xho*I restriction enzymes were used to linearize the pJRoC30 plasmid. The linearized vector was loaded onto a preparative agarose gel and purified with the NucleoSpin Gel and PCR Clean-up kit. *Mwe*UPO-1 gene (200 ng) was mixed with the linearized plasmid (100 ng) and transformed into *S. cerevisiae* for in vivo gene reassembly and cloning using a yeast transformation kit. The plasmid was recovered with a Zymoprep yeast plasmid miniprep kit. Since the products of the Zymoprep were impure and the DNA extracted was very low concentrated, the shuttle vector was transformed into supercompetent *E. coli* XL2-Blue cells and plated onto LB-ampicillin plates. Single colonies were grown in 5 mL of LB-ampicillin medium and incubated overnight at 37 °C and 225 rpm. The plasmid was extracted (NucleoSpin plasmid kit), sent for DNA sequencing (GATC Biotech-Eurofins, Luxembourg), and transformed into *S. cerevisiae* for production in a microtiter plate and flask.

### 5.4 Production of *Mwe*UPO-1 in *S. cerevisiae*

Culture media: sterile minimal medium (SC) contained 100 mL of 19.2 g/L filtered yeast synthetic drop-out medium supplement without uracil, 100 mL of 6.7% filtered yeast nitrogen base, 100 mL of filtered 20% raffinose, 700 mL of ddH_2_O and 1 mL of 25 g/L filtered chloramphenicol. SC drop-out plates contained 100 mL 19.2 g/L filtered yeast synthetic drop-out medium supplement without uracil, 100 mL 6.7% filtered yeast nitrogen base, 20 g autoclaved bacto-agar, 100 mL 20% filtered glucose, 1 mL 25 g/L filtered chloramphenicol and ddH_2_O to 1,000 mL. The sterile expression medium contained 720 mL of autoclaved YP, 111 mL of 20% filtered galactose, 67 mL of 1 M filtered KH_2_PO_4_ pH 6.0 buffer, 31.6 mL of absolute ethanol, 22 mL of filtered MgSO_4_ 0.1 M, 1 mL of 25 g/L filtered chloramphenicol, and ddH_2_O to 1000 mL. YP medium contained 10 g of yeast extract, 20 g of peptone, and ddH_2_O to 650 mL. YPD solution contained 10 g of yeast extract, 20 g of peptone, 100 mL of 20% sterile glucose, 1 mL of 25 g/L chloramphenicol, and ddH_2_O to 1,000 mL. The Luria–Bertani (LB) medium was prepared with 5 g of yeast extract, 10 g of peptone, 10 g of NaCl, 100 mg of ampicillin/25 mg of zeocine, and ddH_2_O to 1000 mL.

#### 5.4.1 Microplate Expression

Individual colonies of *Mwe*UPO-1 were picked and cultured in sterile 96-well plates containing 50 μL of SC minimal medium. In each plate, well H1 was not inoculated (culture media control). Plates were sealed to prevent evaporation and incubated at 30 °C, 225 rpm, and 80% relative humidity in a humid shaker (Minitron-INFORS; Biogen, Spain). After 48 h, 150 μL of expression medium was added to each well, followed by culture for an additional 48 h at 25 °C. The plates were centrifuged at 2,000 rpm (at 4 °C) for 20 min, and finally, 20 μL portions of the supernatants were screened for activity with DMP.

#### 5.4.2 Small-Scale Flask Fermentation

A single colony was picked from an SC drop-out plate, inoculated in a flask with 10 mL of SC minimal medium, and incubated for 48 h at 30 °C and 230 rpm. An aliquot of cells was used to inoculate a minimal medium (10 mL) in a 100 mL flask (OD_600_ = 0.25). The cells completed two growth phases (6–8 h), and then, the expression medium (9 mL) was inoculated with the preculture (1 mL) (OD_600_ of 0.1). After incubating for 72 h at 25 °C and 230 rpm (maximal UPO activity; OD_600_ = 25–30), the cells were pelleted by centrifugation at 5000 rpm for 20 min (at 4 °C) and the supernatant was used for activity assays.

### 5.5 Cloning of *Mwe*UPO-1 in *P. pastoris*

*Mwe*UPO-1 was cloned into the vector pBSY5Z and produced using the carbon source repressed promoter PDF as described before.^34,35^

### 5.6 Production of *Mwe*UPO-1 Expressed by *P. pastoris* in Fed-Batch Bioreactor

*Mwe*UPO-1 *P. pastoris* clone was cultivated in a 5 L glass vessel bioreactor (F1, BIONET, Spain). The basal salts medium (BMG) for the bioreactor with an initial volume of 1.7 L contained 26.7 mL/L 85% phosphoric acid, 0.93 g/L CaSO_4_·2H_2_O, 14.9 g/L MgSO_4_·7H_2_O,18.2 g/L K_2_SO_4_, 4.13 g/L KOH, and 40 g/L glycerol. The PTM_1_ Trace salts solution was sterilized by filtration and consists of the following ingredients: 6 g/L CuSO_4_ 5H_2_O, NaI 0.08 g/L, 3 g/L MnSO_4_ H_2_O, 0.2 g/L Na_2_MoO_4_, 0.02 g/L H_3_BO_3_, 0.5 g/L CoCl_2_, 20 g/L ZnCl_2_, 65 g/L FeSO_4_ 7H_2_O, 0.2 Biotin, 5 mL/L sulfuric acid. After autoclaving the bioreactor with the media, 4.35 mL/L PTM_1_ trace salts and 1 mL of Antifoam B (Sigma-Aldrich, Germany) were added. The pH was adjusted to 5.0 with ammonium hydroxide solution (28%) and was kept constant during cultivation. The fermentation process was started by adding 120 mL of *P. pastoris* preculture grown on BMG medium in a 500 mL Erlenmeyer shaking flask at 180 rpm and 30 °C for 18 h. The glycerol batch was run at 800 rpm and 30 °C. After the glycerol had been completely consumed, the glycerol fed-batch phase followed with the addition of 50% (w/v) glycerol feed, containing 12 mL/L of PTM1 trace salt (dissolved oxygen (DO) concentration should be kept above 20–30%). After completion of the glycerol fed-batch phase, indicated by DO peak after 18–24 h, the methanol feed started by adding 3.6 mL/h per liter methanol, containing 12 mL/L PTM1 trace salt. Within the first two to three hours, the addition of methanol was slowly increased so that the culture could adapt and the DO spike stayed above 80%. Samples were taken regularly. After methanol is consumed or no increase in enzyme activity can be noticed, the vessel is harvested. Culture of *P. pastoris* cells was clarified by centrifugation at 8000 rpm for 20 min at 4 °C. Subsequently, the supernatant was concentrated by tangential ultrafiltration (Pellicon; Millipore, Temecula, CA, US) through a 10 kDa-pore-size membrane (Millipore) by means of a peristaltic pump (Masterflex Easy Load; Cole-Parmer, Vernon Hills, IL).

### 5.7 Purification of *Mwe*UPO-1 Produced by *P. pastoris*

Recombinant *Mwe*UPO-1 purification was carried out by anion exchange chromatography (KTA purifier, GE Healthcare, WI, USA). The concentrated sample was filtered and dialyzed in 20 mM bis–tris buffer pH 7.0 (buffer A). The sample was loaded onto a strong anion-exchange column (HiTraP QFF, Amersham Bioscience) pre-equilibrated with buffer A. The proteins were eluted with a linear gradient with buffer B (20 mM bis–tris buffer pH 7.0 containing 1 M NaCl) in two phases at a flow rate of 1 mL min^–1^: from 0 to 20% over 40 min and from 20 to 100% over 10 min. Fractions with *Mwe*UPO-1 activity were pooled, concentrated, dialyzed against buffer A, and further purified by 10 μm high-resolution anion exchange Biosuite Q (Waters, MA, USA) pre-equilibrated with buffer A. The proteins were eluted again with a linear gradient with buffer B in two phases at a flow rate of 1 mL min^–1^: from 0 to 20% over 40 min and from 20 to 100% over 10 min. The fractions with *Mwe*UPO-1 activity were pooled, dialyzed against 20 mM bis–tris buffer pH 7.0, concentrated, and stored at 4 °C.

### 5.8 Biochemical Characterization

ABTS kinetic constants were estimated in sodium phosphate/citrate buffer (pH 4.0, 100 mM), DMP kinetic constants in phosphate buffer (pH 6.0, 100 mM), and veratryl alcohol and H_2_O_2_ in phosphate buffer (pH 7.0, 100 mM). All kinetics except for H_2_O_2_ contained a fixed concentration of H_2_O_2_ (2 mM). H_2_O_2_ kinetic constants were estimated using veratryl alcohol (2.5 mM) as a reducing substrate. Reactions were performed in triplicate using 96 well microplates and final volumes of 0.2 mL. All reactions were initiated by the addition of 180 μL of a reagent mixture containing buffer, substrate, and H_2_O_2_ at the expected concentrations on the microplate wells containing 20 μL of the purified enzyme, conveniently diluted in reaction buffer. Substrate oxidations were followed through spectrophotometric changes (ε_418_ ABTS^•+^ = 36,000 M^–1^ cm^–1^; ε_469_ cerulignone = 27,500 M^–1^ cm^–1^, ε_310_ veratraldehyde = 9300 M^–1^ cm^–1^). To calculate the *K*_m_ and *k*_cat_ values, the average *V*_max_ was represented against substrate concentration and fitted to a single rectangular hyperbola function with SigmaPlot 10.0, where parameter a was equal to *k*_cat_ and parameter b was equal to *K*_m_.

pH activity profile with veratryl alcohol was calculated with appropriate dilutions of enzyme samples, prepared in such a way that aliquots of 20 μL gave rise to a linear response in kinetic mode. The optimum pH activity was determined using Britton and Robinson buffer (100 mM) at different pH values (from 2.0 to 9.0), veratryl alcohol (5 mM) and H_2_O_2_ (2 mM). The activities were measured in triplicate, and the relative activity (in percent) is based on the maximum activity at a certain pH for each enzyme.

The kinetic thermostability of *Mwe*UPO-1 was estimated by assessing its *T*_50_ values using 96-well gradient thermocyclers (Mycycler, Bio-Rad, USA). Appropriate UPO dilutions were prepared with the help of the robot in such a way that 20 μL aliquots gave rise to a linear response in the kinetic mode. Then, 50 μL were used for each point in the gradient scale and a temperature gradient profile ranging from 20 to 80 °C was established as follows (in °C): 20.0, 30.0, 31.6, 34.6, 39.5, 45.3, 49.6, 52.8, 55.0, 56.9, 59.9, 64.3, 69.7, 75.0, 78.1, and 80.0. After 10 min of incubation, samples were chilled out on ice for 10 min and further incubated at RT for 5 min. Afterward, 20 μL of samples were assayed in sodium phosphate (pH 6.0, 100 mM), containing H_2_O_2_ (2 mM) and DMP (2 mM). Reactions were performed in triplicate, and substrate oxidations were followed through spectrophotometric changes. The thermostability values were deduced from the ratio between the residual activities incubated at different temperature points and the initial activity at RT.

### 5.9 Enzymatic Reactions

Experimental conditions were set up according to the previously reported with the enzymes *Mro*UPO for alkanes,^14^ fatty acids,^15^ and isophorone oxidation,^26^*Mwe*UPO for prednisone oxidation,^8^ and *Cgl*UPO for testosterone oxidation,^25^ with slight modifications. Reactions were carried out in a final volume of 5 mL using substrate concentrations of 0.3 mM for alkanes (dodecane and tetradecane), 0.1 mM for fatty acids (lauric and myristic acids), 0.1 mM for terpenoids (*R*-limonene and isophorone), and 0.5–2 mM for steroids (testosterone and prednisone). Reaction mixtures were prepared in sodium phosphate buffer 50 mM (pH 5.5) containing 2.5 mM H_2_O_2_, 0.5–1.0 μM purified *Mwe*UPO-1, and the corresponding substrate. Prior to use, the substrates were dissolved in acetone and added to the buffer to reach a final acetone concentration of 20% (v/v). The testosterone reactivity was additionally tested in 5% (v/v) of both acetone and acetonitrile. Control experiments were performed simultaneously under the same experimental conditions but in the absence of an enzyme. Reactions were incubated for 1–3 h at 40 °C (alkanes) or 30 °C (rest of the substrates) and 200 rpm. Products were recovered by liquid–liquid extraction with methyl *tert*-butyl ether (alkane and fatty acid reactions) or ethyl acetate (steroids and terpenoids reactions) and dried under a N_2_ atmosphere. Dried samples from alkane and fatty acid reactions were suspended in 100 μL of pyridine (anhydrous) and 100 μL of *N,O*-bis(trimethylsilyl)trifluoroacetamide (BSTFA) was used to prepare the trimethylsilyl (TMS) derivatives that were analyzed by GC-MS. To ensure the complete silylation, the mixture was heated at 70 °C for 30 min in glass vials.

### 5.10 GC-MS Analyses

Chromatographic analyses were performed with Shimadzu GC-MS QP2020 Ultra equipment, using a fused-silica SH-Rxi-5MS capillary column (30 m × 0.25 mm internal diameter, 0.25 μm film thickness) from Shimadzu. The oven was heated from 50 °C (1.5 min) to 90 °C (2 min) at 30 °C·min^–1^ and then from 90 to 250 °C (15 min) at 8 °C·min^–1^. The injection was performed at 250 °C and the transfer line was kept at 300 °C. Compounds were identified by mass fragmentography as previously reported^14,15^ and comparing their mass spectra with those of the Wiley and NIST libraries, and authentic standards. The injection volume was 2 μL with a split ratio of 1:10 and an injection temperature of 250 °C.

### 5.11 HPLC Analyses

Analysis of prednisone and its oxidation products was performed on an HPLC Shimadzu I-Series-Plus equipped with a diode array detector. Separation of products was carried out using a reverse phase column, Shim-pack GWS C18 (5 μm, 150 mm × 4.6 mm, Shimadzu). The column was eluted at a flow rate of 1.0 mL min^–1^ and 40 °C with aqueous 0.1% (v/v) formic acid/ACN, 95:5 for 5 min, followed by a 15 min linear gradient to 100% ACN. Reactions performed with testosterone as a substrate were analyzed using the same HPLC system equipped with an additional evaporative light scattering detector ELSD-LTII (Shimadzu), operating at 60 °C, Gain 6, and a nitrogen pressure of 230 kPa. Separation was performed with aqueous 0.1% (v/v) TFA/ACN, 80:20 for 1 min, followed by a 20 min linear gradient to 55% ACN, and a second 3 min linear gradient to 90% ACN. The final level was maintained until all analytes had been eluted from the column (flow rate 1.0 mL min^–1^, column temperature 30 °C).

### 5.12 Crystallization and Structure Determination of *Mwe*UPO-1 and Its Complexes

*Mwe*UPO-1 was heterologously expressed by *P. pastoris*, purified to homogeneity, and then deglycosylated with Endo H, after which the sample was concentrated with a 10 kDa filtration membrane to 11 mg mL^–1^ in 50 mM sodium citrate pH 5.6 buffer. For the screening of crystallization conditions, several commercial crystallization kits were tested using an Oryx8 robot (Douglas Instruments) for microbatch screening and optimization in 96-well crystallization plates (Hampton Research). Specifically, JBS Classic and PACT (Jena Bioscience) were used. Crystals were grown by the sitting-drop vapor diffusion method at 18 °C. After 48 h, many crystals were obtained in polyethylene glycol (PEG) based conditions, the best plates growing from 10 to 20% (v/v) PEG 4000, 0.2 M (NH_4_)_2_SO_4_, and a protein:reservoir ratio of 1.5:1. Drop sizes were adjusted for each case. For data collection, crystals were cryoprotected with 15–20% (v/v) glycerol previously to be immersed in liquid nitrogen.

Diffraction data were collected in the XALOC beamline at the ALBA synchrotron (Cerdanyola del Valles, Spain). The data were integrated and scaled with XDS^36^ and merged using an AIMLESS program from the CCP4 package.^37^ These crystals were indexed in the *P*4_3_2_1_2 space group, containing two molecules in the asymmetric unit (mol/ASU) and 51% solvent content within the cell. Then, a molecular replacement was carried out with MOLREP using the peroxygenase from *Marasmius rotula*, *Mro*UPO (PDB code 5FUK). Our refined models became the template for the rest of the data sets. For automated rigid body refinement, REFMAC within the CCP4 suite^38^ was used and 5% of structure factor amplitudes were randomly selected in the calculation of free R-factor. Afterward, Coot^39^ was used for manual refinement and model building of the ligands, and at the later stages, *N*-acetylglucosamine and water molecules were added, which, combined with more rounds of restrained refinement, led to the final statistics reported in Supplementary Table S2.

For the *Mwe*UPO-1 complexed structures, nine peroxidative or peroxygenative substrates were used, all of them being dissolved in methanol except prednisone, which was dissolved in 75% DMSO (v/v). Preformed *Mwe*UPO-1 crystals were soaked in the precipitant solution, supplemented with 10 to 40 mM ligand for one to 16 h. As previously performed, all X-ray diffraction data collected at the ALBA synchrotron were processed with XDS and AIMLESS programs. The complexes were solved by Fourier transformations using the coordinates from the unsubstituted form, and substrate ligands, solvent molecules, and waters were fitted into the density with Coot. For isophorone and R-limonene, not described in the Protein Data Bank (PDB), restrain files were generated with grade Web Server (grade.globalphasing.org/) and CSD Mogul in PHENIX program,^40^ respectively. Soaking with *S*-limonene was unsuccessful, showing no density at the active site. Details for the complexes with dodecane, tetradecane, lauric acid, myristic acid, *R*-limonene, testosterone, prednisone, and isophorone are provided in Supplementary Table S2.

Verification of these structures was done via the PDB Validation server (validate.wwpdb.org) before coordinates and associated structure factors were deposited in the Protein Data Bank (PDB). The structural figures were prepared with PyMOL.

### 5.13 Accession Codes

Atomic coordinates and structure factors of *Mwe*UPO-1 and its complexes have been deposited in the Protein Data Bank (PDB), under PDB IDs 8RNJ (Protein), 8RNK (Myristic acid), 8RNL (Lauric acid), 8RNM (Testosterone), 8RNN (Prednisone), 8RNO (Isophorone), 8RNP (*R*-limonene), 8RNQ (Dodecane), and 8RNR (Tetradecane).

### 5.14 Molecular Docking Analysis

The crystal structure of *Mwe*UPO-1 was used as a template for the automatic molecular docking of prednisone into its active site. All water molecules and other heteroatoms were removed from the structure prior to protein preparation. Autodock Tools was used to create a “.pdbqt” file, a PDB-modified file containing the coordinates of the enzyme and the substrate. Docking studies in the heme channel of *Mwe*UPO-1 were carried out using AutoDock Vina^41^ with Ile55, Leu58, Val62, Ile84, Thr152, Ile153, Glu157, and Phe160 defined as flexible residues. The docking space was visually defined using Autodock Tools. The grid dimensions were 36 × 36 × 36 grid points with a grid center at 9.246 × −8.425 × 23.085. Default parameters were defined with an exhaustiveness of 16 and an energy range of 8. Calculations were performed by applying the Lamarckian genetic algorithm method. From the solutions, the best poses of prednisone were chosen based on minimum binding energy. The structural figures were prepared with PyMOL.

## Supporting Information Available

Data of *Mwe*UPO-1 expression levels in *S. cerevisiae* and *P. pastoris*; crystallographic statistics of *Mwe*UPO-1 complexes; physicochemical characterization of the *Mwe*UPO-1; sequence alignments of *Mwe*UPO-1; general catalytic mechanism of UPO; additional crystallographic soakings of *Mwe*UPO-1; mass spectra of the products identified in the GC/MS analysis (PDF)
