# Supplementary material for: Structural Insights and Reaction Profile of a New Unspecific Peroxygenase from Marasmius wettsteinii Produced in a Tandem-Yeast Expression System
Source: ACS Chem Biol. 2024 Oct 5;19(10):2240–53. doi: 10.1021/acschembio.4c00504 (PMC11925332; doi:10.1021/acschembio.4c00504)

Supplementary information for:

Structural insights and reaction profile of a new unspecific peroxygenase from *Marasmius wettsteinii* produced in a tandem-yeast expression system

Israel Sanchez-Moreno[1‡], Angela Fernandez-Garcia[2‡], Ivan Mateljak[3‡], Patricia Gomez de Santos[3], Martin Hofrichter[4], Harald Kellner[4], Julia Sanz-Aparicio[2] and Miguel Alcalde[1*]

[1] Department of Biocatalysis, Institute of Catalysis, CSIC, 28049 Madrid, Spain.

[2] Department of Crystallography & Structural Biology, Institute of Physical Chemistry "Blas Cabrera", CSIC, 28006 Madrid, Spain.

[3] EvoEnzyme S.L., C/ Faraday 7, Parque Científico de Madrid, 28049 Madrid, Spain.

[4] Department of Bio- and Environmental Sciences TU Dresden, International Institute Zittau, Markt 23, Zittau, 02763, Germany.

* Correspondence should be addressed to: M.A. (malcalde@icp.csic.es).

‡ These authors contributed equally to this work.

Supplementary Table S1. Secretion levels of *Mwe*UPO-1 in different formats in *S. cerevisiae* and *P. pastoris.*

	Secretion levels (mg/L)		
	96-well plate	Flask	5L fed-batch bioreactor
S. cerevisiae	5.7	11.5	n.d.
P. pastoris	6.1	48.2	690

n.d: not determined.

Table S2. Crystallographic statistics of *Mwe*UPO-1 complexes.

**Crystal data**	***Mwe*UPO-1**	***Mwe*UPO-1-C12**	***Mwe*UPO-1-C14**	***Mwe*UPO-1-LAU**	***Mwe*UPO-1-MYR**	***Mwe*UPO-1-PDN**	***Mwe*UPO-1-TES**	***Mwe*UPO-1-ISR**	***Mwe*UPO-1-LIM**
Space group	$P\ 4_3\ 2_1\ 2$	$P\ 4_3\ 2_1\ 2$	$P\ 4_3\ 2_1\ 2$	$P\ 4_3\ 2_1\ 2$	$P\ 4_3\ 2_1\ 2$	$P\ 4_3\ 2_1\ 2$	$P\ 4_3\ 2_1\ 2$	$P\ 4_3\ 2_1\ 2$	$P\ 4_3\ 2_1\ 2$
Molecules/a.u.	2	2	2	2	2	2	2	2	2
**Unit cell parameters**									
a, b (Å)	73.57	76.28	76.12	75.40	75.50	76.13	76.0	76.23	75.01
c (Å)	186.67	186.69	186.55	186.25	186.05	186.73	185.83	185.48	186.09
**Data collection**									
Beamline	XALOC (ALBA)	XALOC (ALBA)	XALOC (ALBA)	XALOC (ALBA)	XALOC (ALBA)	XALOC (ALBA)	XALOC (ALBA)	XALOC (ALBA)	XALOC (ALBA)
Temperature (K)	100	100	100	100	100	100	100	100	100
Wavelength (Å)	0.9792	0.9793	0.9793	0.9792	0.9792	0.9793	0.9792	0.9793	0.9793
Resolution (Å)	47.51-1.60 (1.63-1.60)	46.70-1.80 (1.84-1.80)	48.16-1.72 (1.75-1.72)	47.93-1.60 (1.63-1.60)	47.92-1.60 (1.63-1.60)	46.63-1.58 (1.61-1.58)	48.01-1.95 (2.00-1.95)	48.02-2.05 (2.11-2.05)	47.80-1.92 (1.96-1.92)
**Data processing**									
Total reflections	498779 (23812)	329966 (19231)	345349 (18870)	528037 (26196)	476543 (20090)	476290 (24725)	288409 (21429)	223071 (17239)	258635 (15933)
Unique reflections	67994 (3379)	51958 (3003)	57947 (2997)	71809 (3484)	71442 (3506)	74091 (3697)	40573 (2816)	33452 (2627)	39559 (2581)
Multiplicity	7.3 (7.0)	6.4 (6.4)	6.0 (6.3)	7.4 (7.5)	6.7 (5.7)	6.4 (6.7)	7.1 (7.6)	6.7 (6.6)	6.5 (6.2)
Completeness (%)	99.7 (98.2)	99.8 (99.9)	98.4 (97.7)	100.0 (100.0)	99.6 (100.0)	98.0 (99.6)	99.7 (100)	95.2 (97.6)	95.4 (94.2)

Mean I/σ (I)	16.5 (2.8)	22.7 (2.2)	21.0 (3.2)	18.2 (2.8)	18.8 (2.1)	19.4 (3.1)	13.2 (2.8)	16.4 (2.3)	12.7 (1.6)
R_{merge}† (%)	5.5 (69.1)	3.8 (64.4)	4.2 (51.8)	5.4 (58.0)	4.5 (60.1)	4.5 (45.7)	8.2 (57.6)	5.1 (55.8)	6.2 (62.5)
R_{pim}†† (%)	2.2 (28.0)	1.6 (26.9)	1.9 (22.1)	2.1 (22.6)	1.8 (27.2)	1.8 (18.7)	3.3 (22.1)	1.9 (21.4)	2.3 (24.2)
**Refinement**									
R_{work} / R_{free}††† (%)	16.7/19.8	17.4/22.0	18.0/20.1	17.0/19.7	17.0/19.3	18.2/21.8	19.4/22.7	20.2/24.8	19.9/25.6
**Nº of atoms/ average B (Å^2)**									
Macromolecules	3672/25.35	3688/39.08	3694/33.51	3662/24.61	3670/26.08	3696/24.14	3683/31.94	3688/56.44	3698/48.82
Ligands	206/28.08	261/53.97	252/42.81	271/34.78	274/35.24	299/32.03	242/41.97	223/65.85	213/61.44
Solvent	444/33.32	394/44.28	465/38.77	509/34.22	476/34.71	574/33.54	407/35.51	167/53.19	216/48.21
All atoms	4322/26.30	4343/40.45	4411/34.60	4442/26.33	4420/27.58	4569/25.84	4332/32.84	4078/56.82	4127/49.44
**Ramachandran plot (%)**									
Favoured	96.2	95.7	96.2	96.6	96.4	96.1	95.7	96.2	95.7
Outliers	0.43	0.43	0.42	0.42	0.43	0.43	0.65	0.43	0.43
**RMS deviations**									
Bonds (Å)	0.007	0.009	0.006	0.006	0.007	0.006	0.007	0.006	0.006
Angles (°)	1.457	1.483	1.353	1.336	1.394	1.378	1.297	1.294	1.426
**PDB accession codes**	8RNJ	8RNQ	8RNR	8RNL	8RNK	8RNN	8RNM	8RNO	8RNP

Values in brackets are for the hight-resolution shell. †Rmerge = ∑hkl ∑i | Ii(hkl) – [I(hkl)]| / ∑hkl ∑i Ii(hkl), where Ii(hkl) is the ith measurement of reflection hkl and [I(hkl)] is the weighted mean of all measurements. ††Rpim = ∑hkl [1/(N - 1)] 1/2 ∑i | Ii(hkl) – [I(hkl)]| / ∑hkl ∑i Ii(hkl), where N is the redundancy for the hkl reflection. †††Rwork / Rfree = ∑hkl | Fo – Fc | / ∑hkl | Fo |, where Fc is the calculated and Fo is the observed structure factor amplitude of reflection hkl for the working / free (5%) set, respectively.

```
MroUPO       MKLAISSSLIALVSVTTALANSQDVVDFSAHPWKAPGPNDSRGPCPGLNTLANHGFLPRN     60
MweUPO-1     MKLAISSAFIALVSVTTALANSQDVVDFSAHPWKAPGPNDSRGPCPGLNTLANHGFLPRN     60
             *******::***************************************************

MroUPO       GRNISVPMIVKAGFEGYNVQSDILILAGKIGMLTSREADTISLEDLKLHGTIEHDASLSR     120
MweUPO-1     GRNISVPMIVKAGFEGYNVQSDILILAGKVGMLTSREADTISLEDLKLHGTIEHDASLSR     120
             *****************************:******************************

MroUPO       EDVAIGDNLHFNEAIFTTLANSNPGADVYNISSAAQVQHDRLADSLARNPNVTNTDLTAT     180
MweUPO-1     EDVAIGDNLHFNEAIFTTLANSNPGADVYNISSAAQVQHDRLADSVARNPNVTNTDLTAT     180
             *********************************************:**************

MroUPO       IRSSESAFFLTVMSAGDPLRGEAPKKFVNVFFREERMPIKEGWKRSTTPITIPLLGPIIE     240
MweUPO-1     IRSSESAFYLTVMSAGDPLRGEAPKKFVNVFFQEERMPIKEGWKRSTTPINLPLLGPIID     240
             ********:***********************:*****************.:*******:

MroUPO       RITELSDWKPTGDNCGAIVLSPEL      264
MweUPO-1     RITELSDWKPTGDNCGAIVLGPGL      264
             ********************.* *
```

Supplementary Figure S1. *Mro*UPO:*Mwe*UPO-1 alignment. In yellow, differences in signal peptide. In green, first amino acid of mature protein. In cyan, differences in mature protein. Alignment generated by Clustal Omega (https://www.ebi.ac.uk/jdispatcher/msa/clustalo).

```
MweUPO-1         MKLAISSAFIALVSVTTALANSQDVVDFS--AHPWKAPGPNDSRGPCPGLNTLANHGFLP   58
PAP230129_4      MKLAISSSLIALVSVTTALANSQDVVDFGASAHPWKAPGPNDSRGPCPGLNTLANHGFLP   60
MroUPO           MKLAISSSLIALVSVTTALANSQDVVDFS--AHPWKAPGPNDSRGPCPGLNTLANHGFLP   58
PAP230120_3      MKLAISSSLIALVSVTTALANSQDVVDFGASAHPWKAPGPNDSRGPCPGLNTLANHGFLP   60
                 *******::*******************.  *****************************

MweUPO-1         RNGRNISVPMIVKAGFEGYNVQSDILILAGKVGMLTSREADTISLEDLKLHGTIEHDASL   118
PAP230129_4      RNGRNISVPMIVKAGFEGYNVQSDVLITAGKVGMLTSREADTISLEDLKLHGTIEHDASL   120
MroUPO           RNGRNISVPMIVKAGFEGYNVQSDILILAGKIGMLTSREADTISLEDLKLHGTIEHDASL   118
PAP230120_3      RNGRNISVPMIVKAGFEGYNVQSDILILAGKIGMLTSREADTISLEDLKLHGTIEHDASL   120
                 ************************:** ***:****************************

MweUPO-1         SREDVAIGDNLHFNEAIFTTLANSNPGADYYNISSAAQVQHDRLADSVARNPNVTNTDLT   178
PAP230129_4      SREDAAIGDNLHFNEAIFTTLANSNPGADVYNISSAAQVQHDRLADSLARNPNVTNTDVT   180
MroUPO           SR-DVAIGDNLHFNEAIFTTLANSNPGADYYNISSAAQVQHDRLADSLARNPNVTNTDLT   177
PAP230120_3      SREDVAIGDNLHFNEAIFTTLANSNPGADVYNISSAAQVQHDRLADSLARNPNVTNTDLT   180
                 ** *.************************ *****************:**********:*

MweUPO-1         ATIRSSESAFYLTVMSAGDPLRGEAPKKFVNVFFQEERMPIKEGWKRSTTPINLPLLGPI   238
PAP230129_4      ATIRASESAFYLTVMSAGDPLRGEAPKKFVNVCFREERMPVKEGWKRSTTPINIPLLVPI   240
MroUPO           ATIRSSESAFFLTVMSAGDPLRGEAPKKFVNVFFREERMPIKEGWKRSTTPITIPLLGPI   237
PAP230120_3      ATIRSSESAFFLTVMSAGDPLRGEAPKKFVNVFFREERMPIKEGWKRSTTPITIPLLGPI   240
                 ****:*****:********************* *:*****:***********.:*** **

MweUPO-1         IDRITELSDWKPTGDNCGAIVLGPGL----------------------------------   264
PAP230129_4      IERIIELSDWKPTGDNCGAIVLSPDLSGGSAWSHPQFEKGGGSGGGSGGSAWSHPQFEKD   300
MroUPO           IERITELSDWKPTGDNCGAIVLSPEL----------------------------------   263
PAP230120_3      IERITELSDWKPTGDNCGAIVLSPELSGGSAWSHPQFEKGGGSGGGSGGSAWSHPQFEKD   300
                 *:** *****************.* *

MweUPO-1         ------------------------- 264
PAP230129_4      GGSGGGSTSRDHMVLHEYVNAAGIT 325
MroUPO           ------------------------- 263
PAP230120_3      GGSGGGSTSRDHMVLHEYVNAAGIT 325
```

Supplementary Figure S2. Alignment of *Mro*UPO, *Mwe*UPO-1 (current work), *Mro*UPO (PAP230120_3) and *Mwe*UPO (PAP230129_4) from[16]. Alignment generated by Clustal Omega (https://www.ebi.ac.uk/jdispatcher/msa/clustalo).

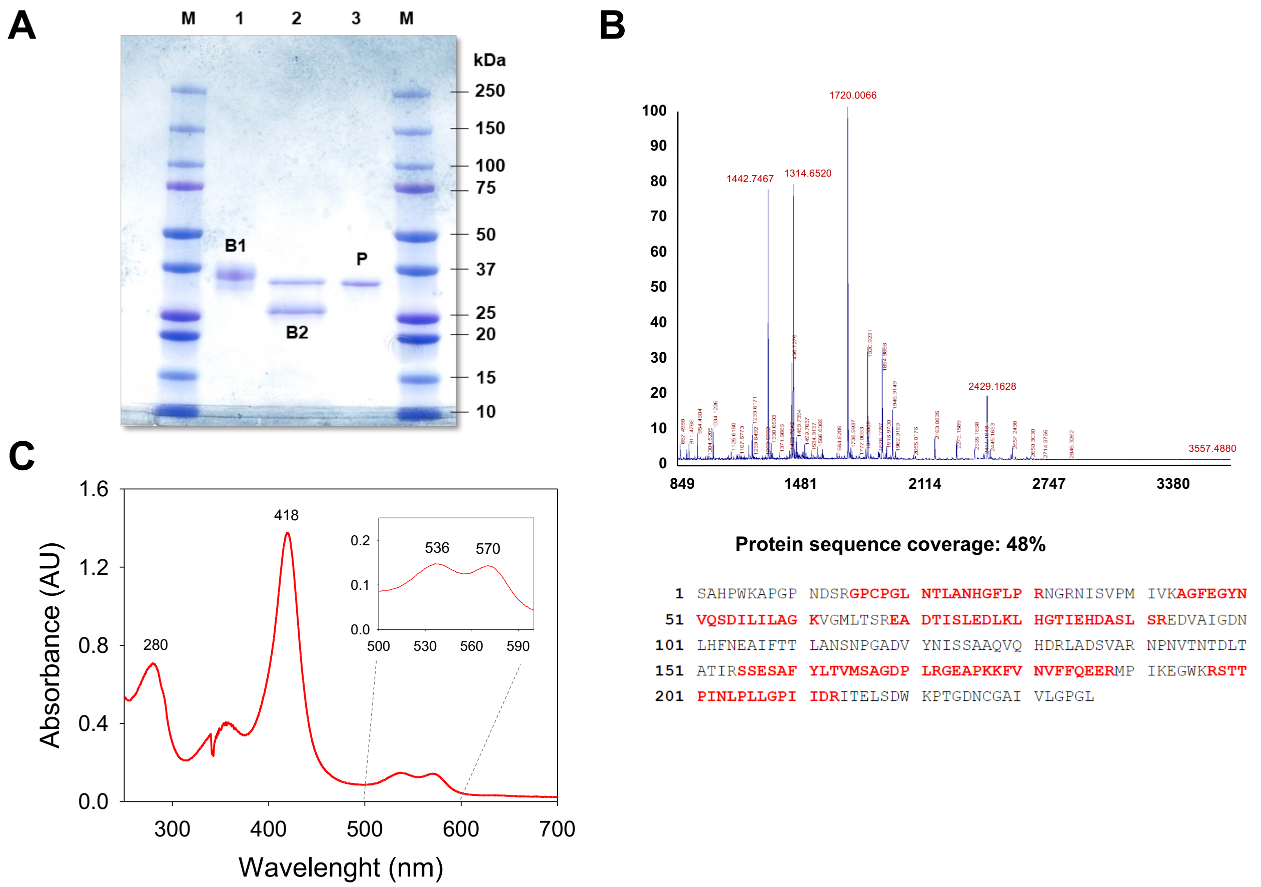

Supplementary Figure S3. Physicochemical characterization of the *Mwe*UPO-1. (**A**) SDS PAGE analysis of the pure *Mwe*UPO-1 before (lane 1, band B1) and after deglycosylation (lane 2, band B2) by digestion with the enzyme PNGase F (lane 3, band P). (**B**) Peptide mass fingerprint analysis of *Mwe*UPO-1. The red shaded sequence indicates the region covered by the peptides identified after tryptic digestion. (**C**) Soret-band Uv-vis spectrum of *Mwe*UPO-1. Insert shows the charge transference bands CT1 (536 nm) and CT2 (570 nm).

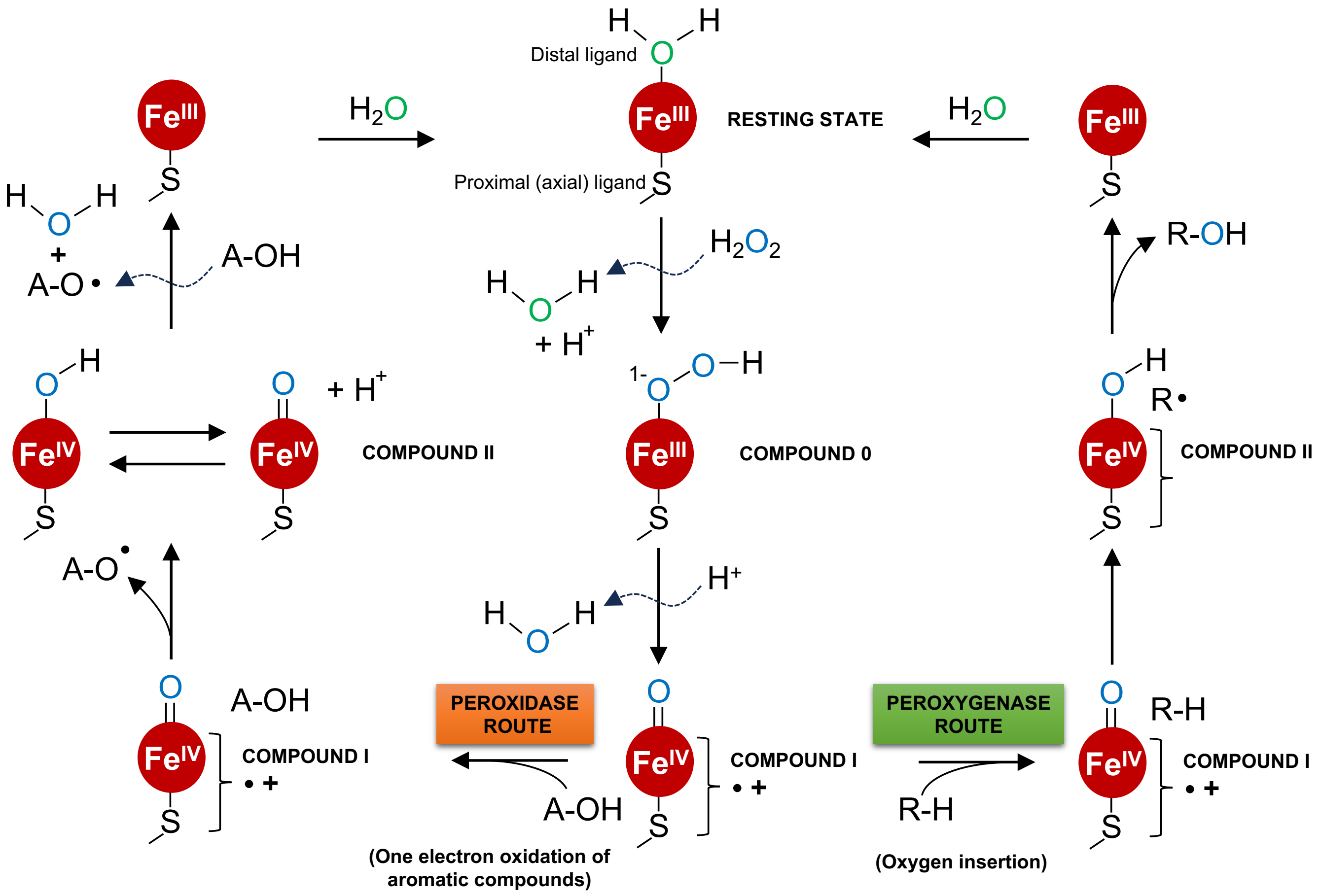

Supplementary Figure S4. General catalytic mechanism of UPO. The dual catalytic cycle of unspecific peroxygenase combines the classic heme peroxidase cycle with the "peroxide shunt" pathway of P450s. Starting from UPO at its resting heme ferric state (Heme-Fe^{3+}←H_2O), H_2O_2 enters to the heme and replaces the molecule of H_2O that was acting as distal ligand leading to the formation of a pre-Compound 0 (Heme-Fe^{3+}←H_2O_2). This intermediate is deprotonated *via* a conserved glutamic acid of the catalytic acid-base pair to form Compound 0, which decays under electron re-arrangement into Compound I, a reactive oxo ferryl cation radical complex ($^{+\bullet}$Heme-Fe^{4+}=O). Compound I can then undergo into two different routes: peroxygenase (mono(per)oxygenase) and peroxidase. During the peroxygenase route (*i.e.* two electron oxidation), a hydrogen (H^+ and e^-) is abstracted from the substrate (R-H) resulting in the protonated ferryl hydroxide complex (Compound II, Heme-Fe^{4+}-OH) and the substrate radical ($R^\bullet$ that stays near the oxygen). Substrate radical and Compound II react immediately with each other to form the hydroxylated product while water binds again as distal heme ligand so the catalytic cycle starts again. In the case of epoxidation reactions, the cycle is slightly modified and Compound II transitionally binds the substrate as a radical via the ferryl oxygen, forming an alkoxy radical complex so there is no H abstraction. In the peroxidase cycle, both Compound I and deprotonated Compound II (in equilibrium with its protonated counterpart) may abstract single electrons (and the corresponding protons) from two substrate molecules (A-OH), which are then released as radicals and may undergo spontaneous coupling or disproportionation reactions.

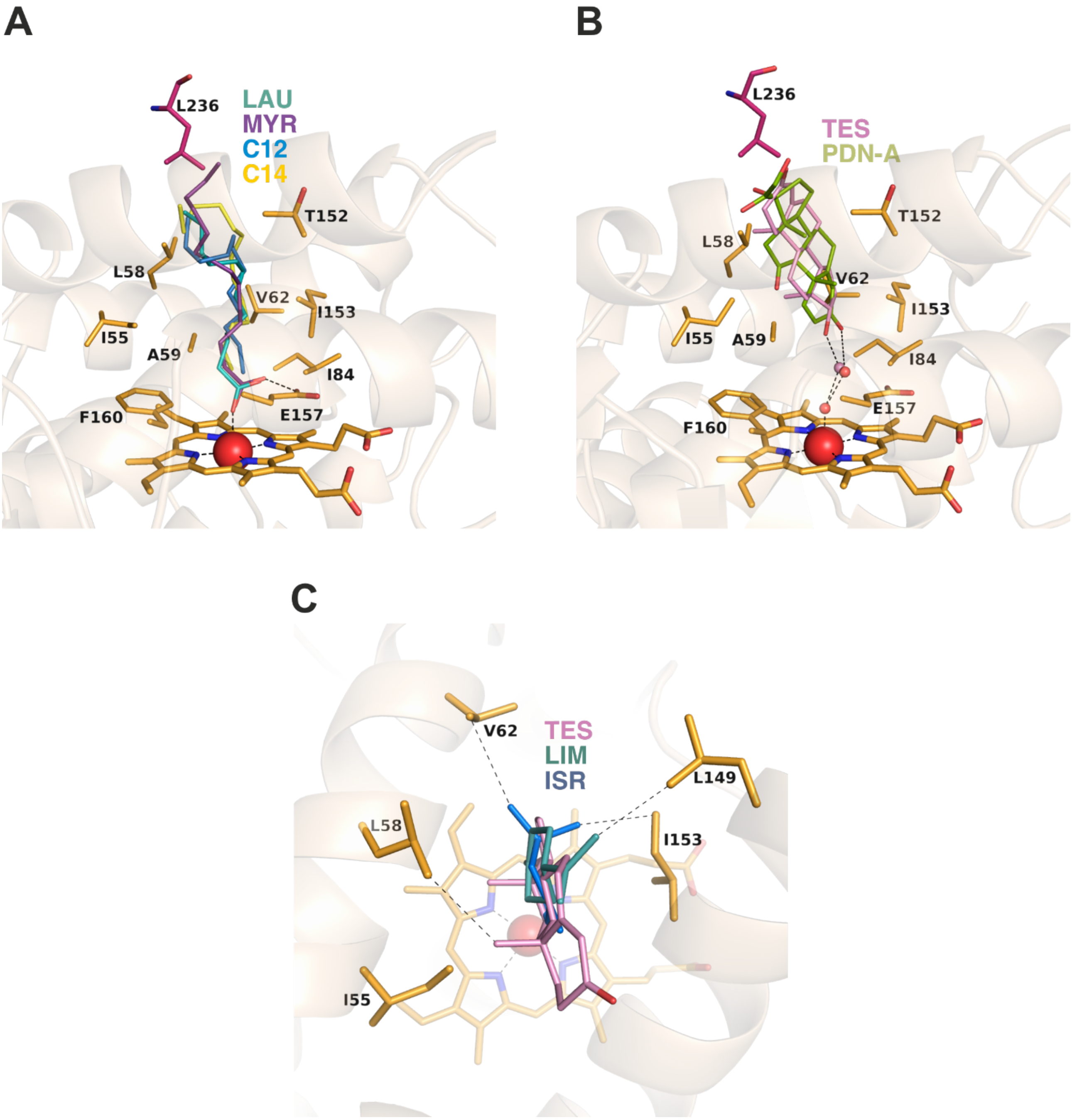

Supplementary Figure S5. Comparison of binding modes and interactions of the captured ligands in the active site of *Mwe*UPO-1. (**A**) Binding of alkanes (dodecane, C12, and tetradecane, C14) and fatty acids (lauric acid, LAU, and myristic acid, MYR, in its extended form) in the heme channel. The polar interactions of the fatty-acids are represented as dashed lines. Leu236 from the adjacent subunit is represented as pink sticks. (**B**) Binding of steroid ketones testosterone and prednisone (TES and PDN-A) in the heme access channel. Water molecules involved in substrate recognition are included as spheres (the color corresponds to each ligand). (**C**) Binding of testosterone (TES), isophorone (ISR) and R-limonene (RMO) in the heme access channel. Hydrophobic interactions shorter than 4 Å are illustrated as dashed lines, with relevant residues contouring the heme channel represented as sticks (in orange).

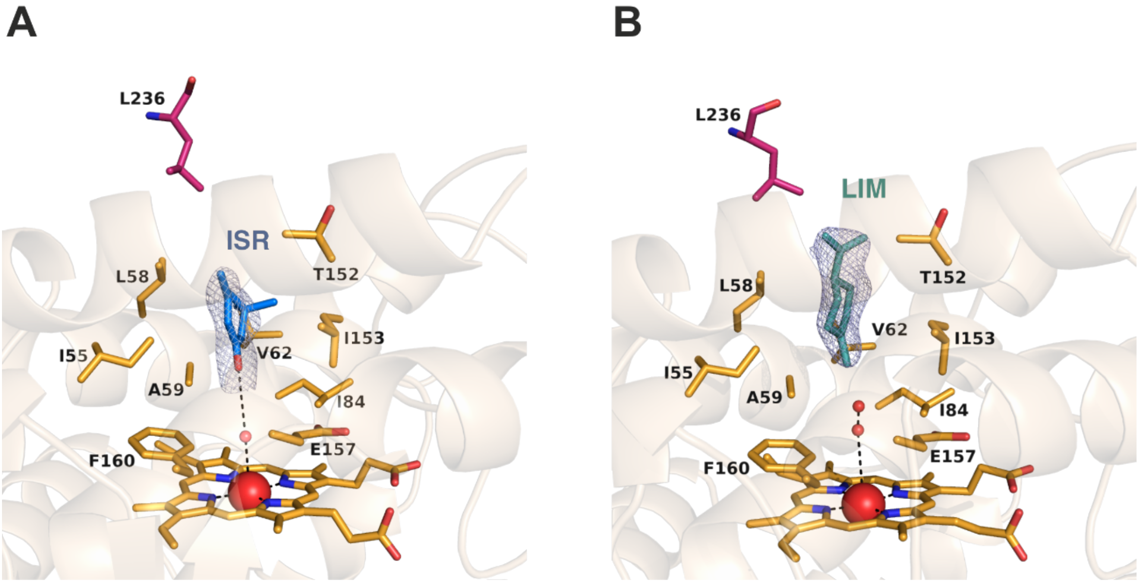

Supplementary Figure S6. Crystallographic structure of the *Mwe*UPO-1 complexes with (**A**) isophorone (ISR) and (**B**) R-limonene (LIM). The relevant residues defining the heme channel are represented as sticks (in orange) and polar bonds are shown as dashed lines. Leu236 from the adjacent subunit is represented in pink sticks. Water molecules involved in substrate recognition are included as red spheres.

Supplementary Data: Mass spectra of the products identified in the GC/MS analysis of the *Mwe*UPO-1 reaction with dodecane, lauric acid, tetradecane, and myristic acid, which were labeled in **Figure 4**. The most representative fragmentations for the molecules identification are indicated with red-dashed arrows.

1) Dodecane (Alk C12):

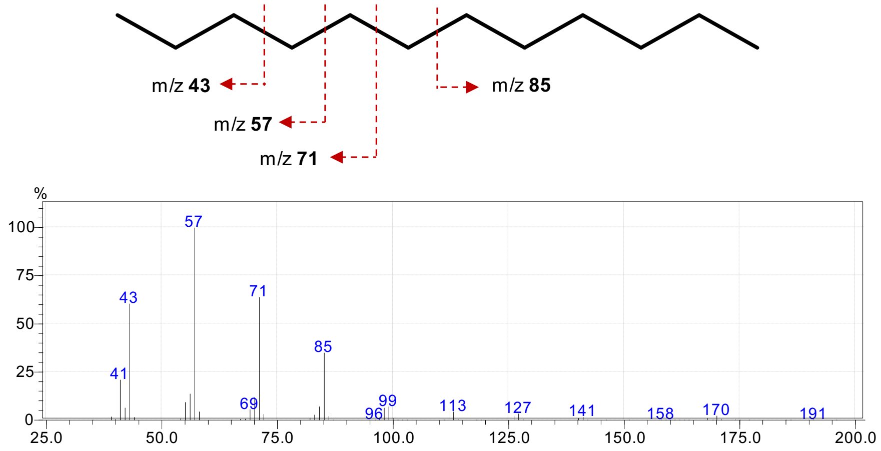

2) 2-keto (C12):

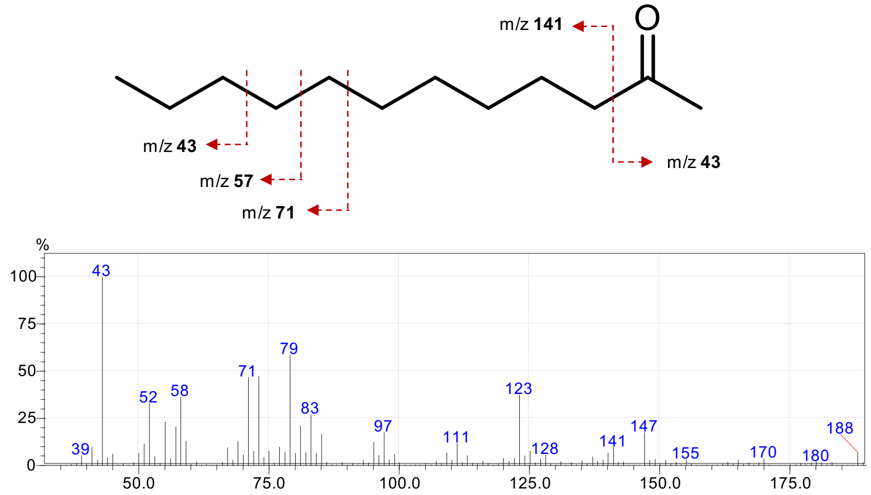

3) COOH (C12) (Lauric acid):

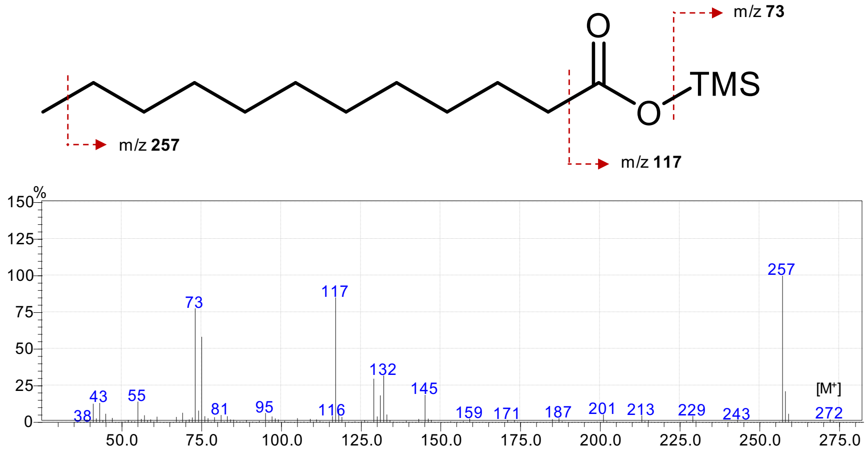

4) 2-keto-11-OH (<u>C12</u>):

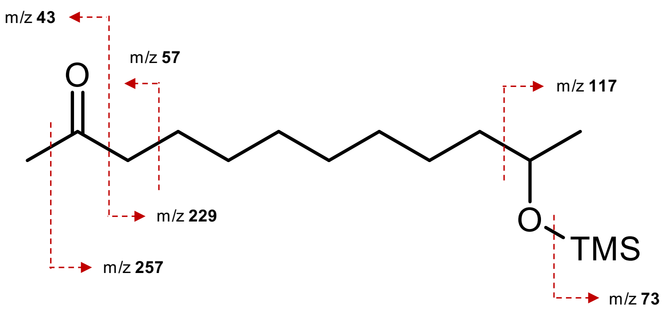

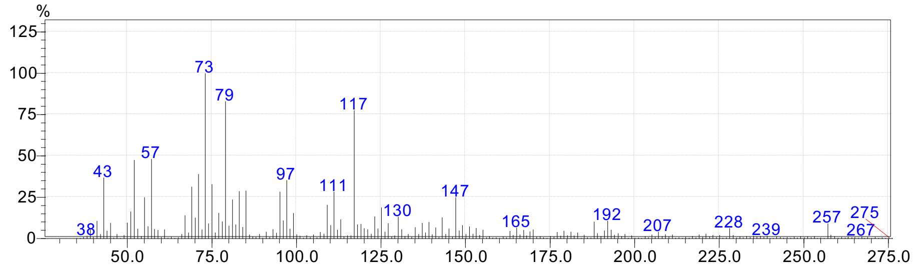

5) (ω-2)-keto-COOH (<u>C12</u>):

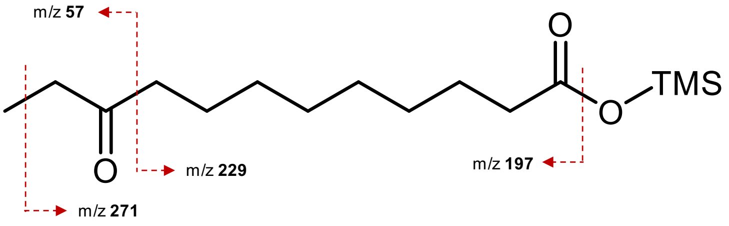

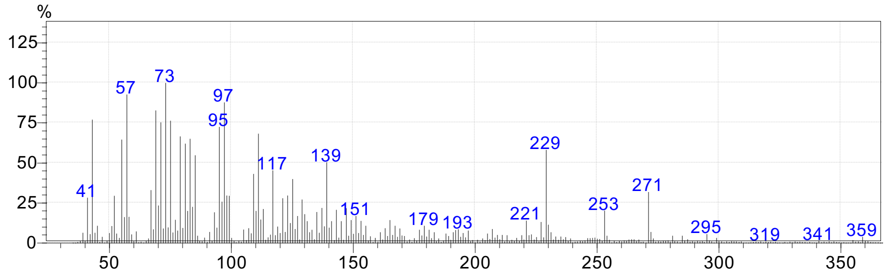

6) 3,4-OH-COOH (<u>C12</u>):

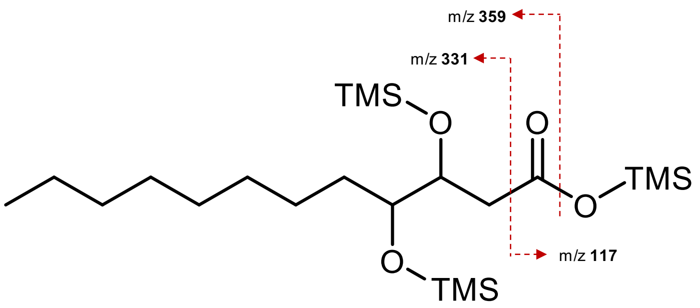

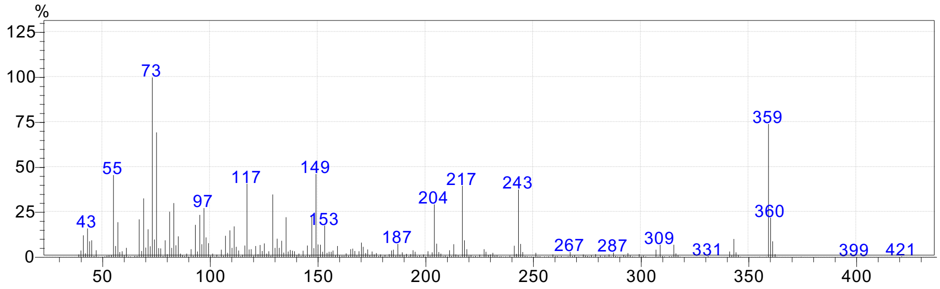

7) α-OH-COOH (<u>C12</u>):

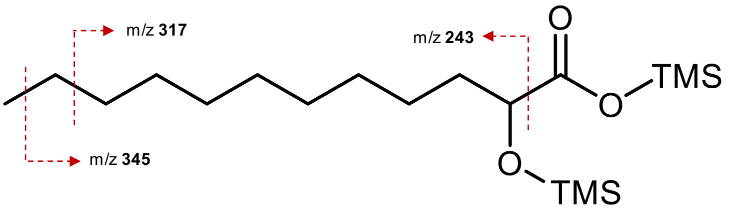

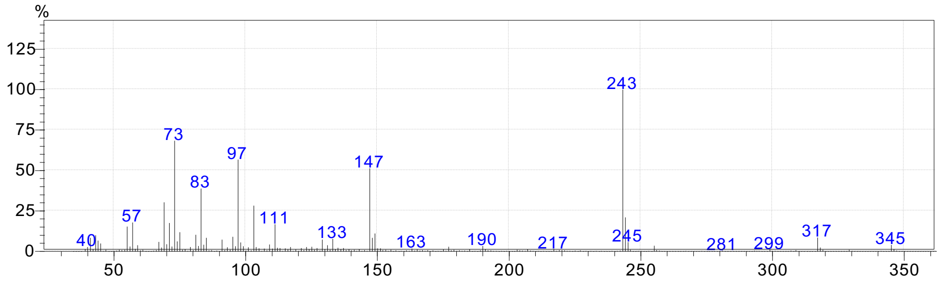

8) (ω-2)-keto-COOH (<u>C12</u>):

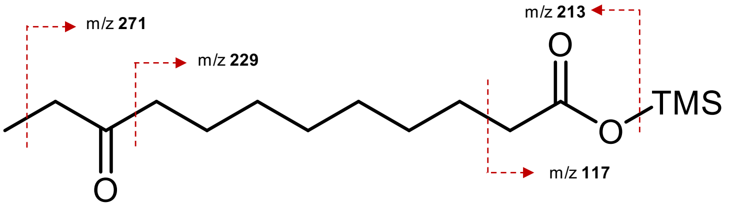

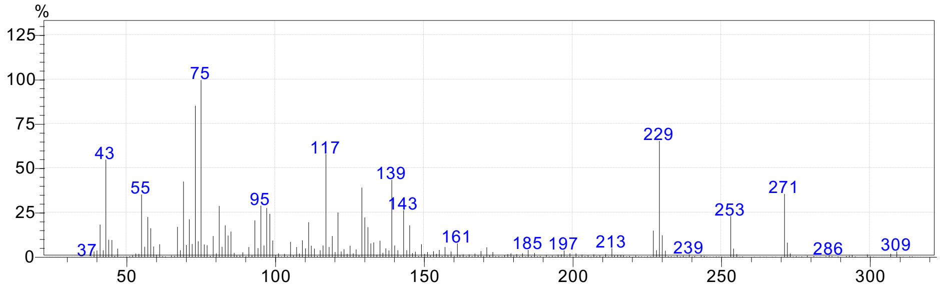

9) (ω-2)-OH-COOH (<u>C11</u>):

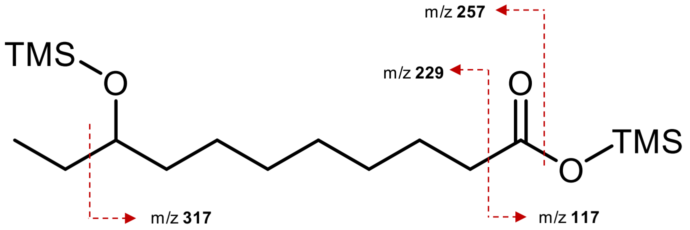

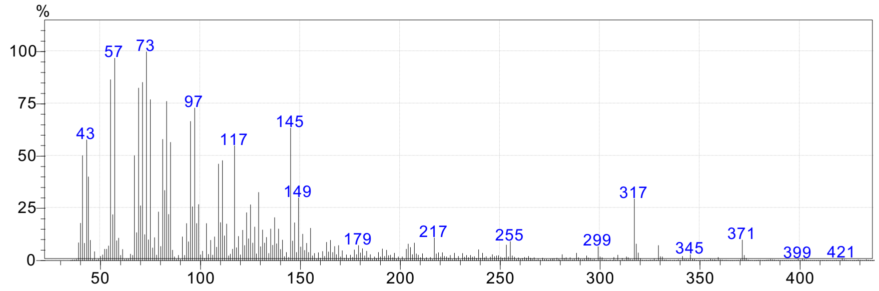

10) (ω-2)-OH-COOH (<u>C12</u>):

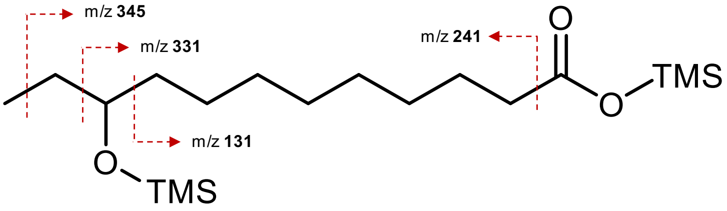

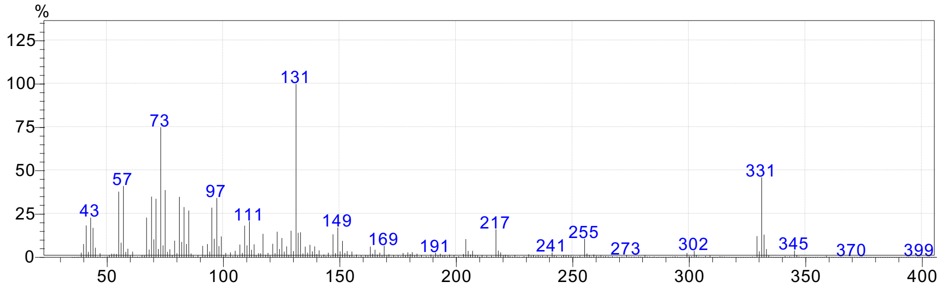

11) (ω-1)-OH-COOH (<u>C12</u>):

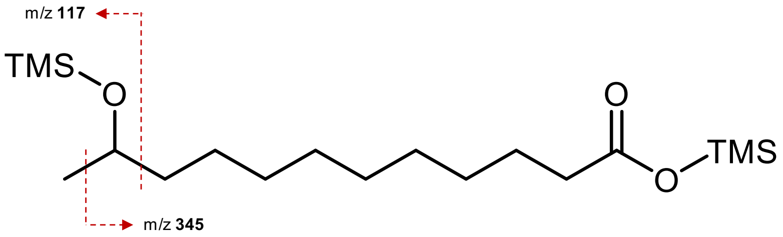

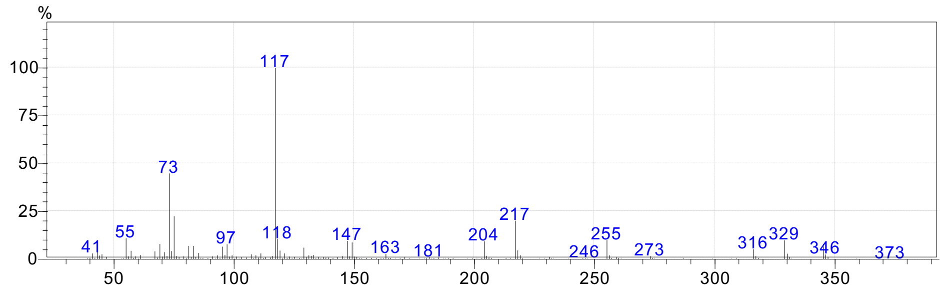

12) (ω)-OH-COOH (<u>C12</u>):

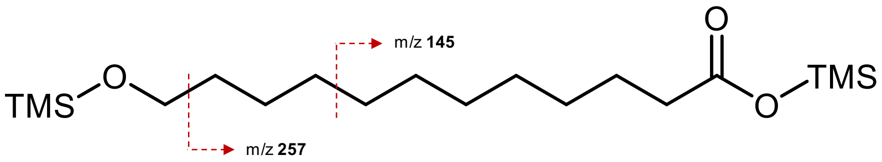

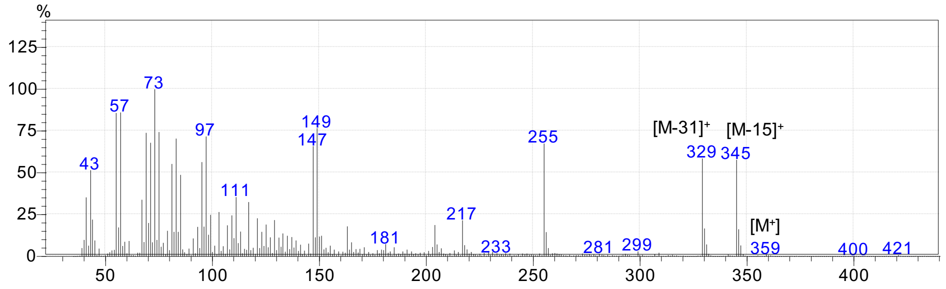

13) di-COOH (<u>C12</u>):

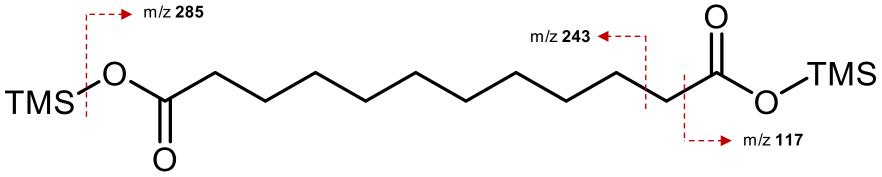

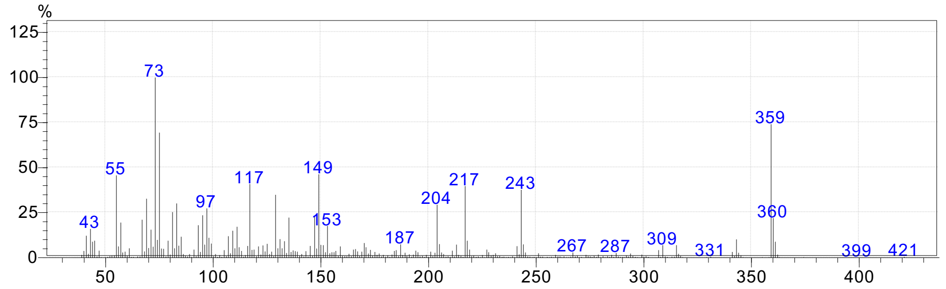

14) COOH (<u>C11</u>):

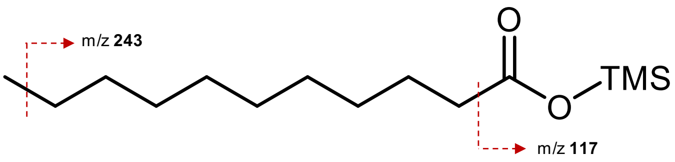

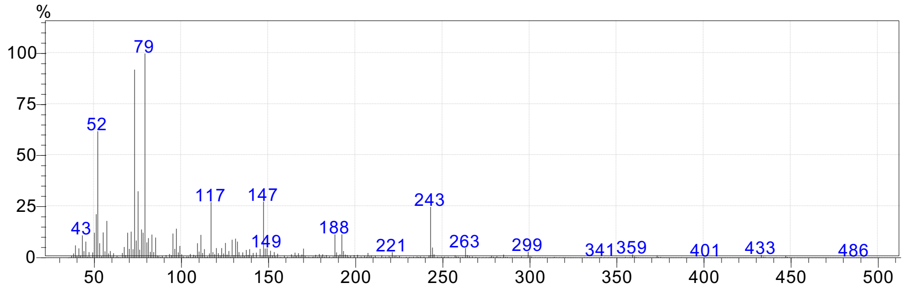

15) 2-keto-4,5-OH (<u>C12</u>):

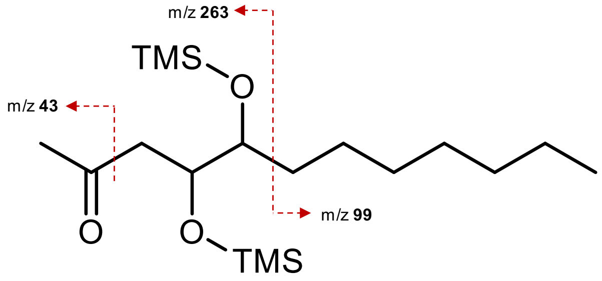

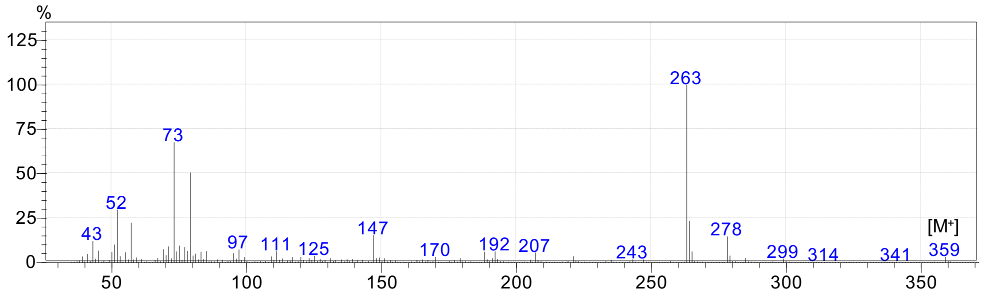

16) 2,10-keto-3,11-OH (<u>C12</u>):

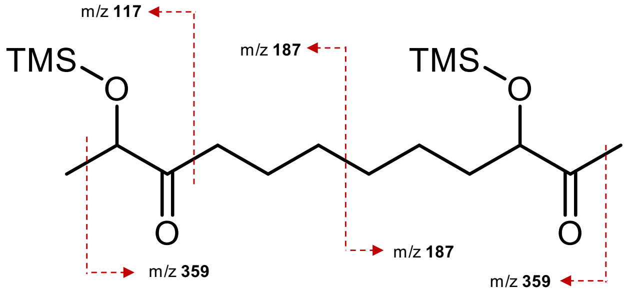

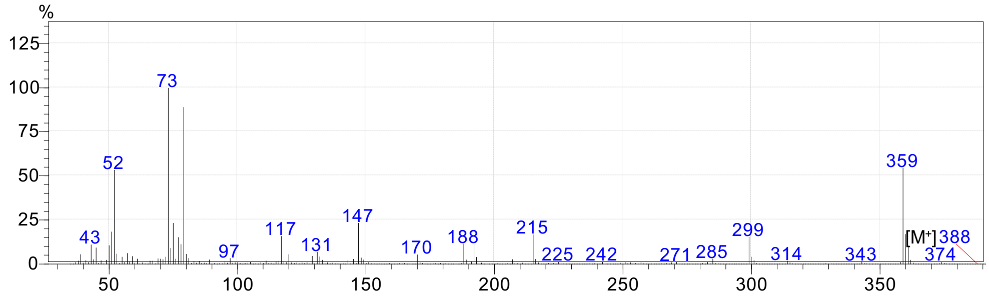

17) 2,11-keto-2,10-OH (<u>C12</u>):

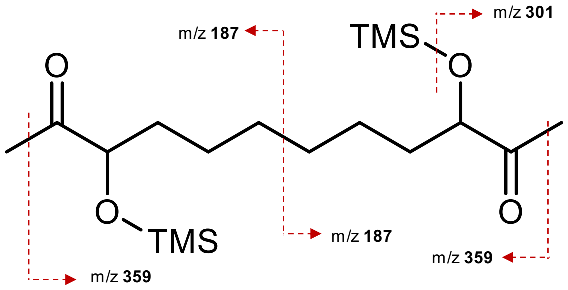

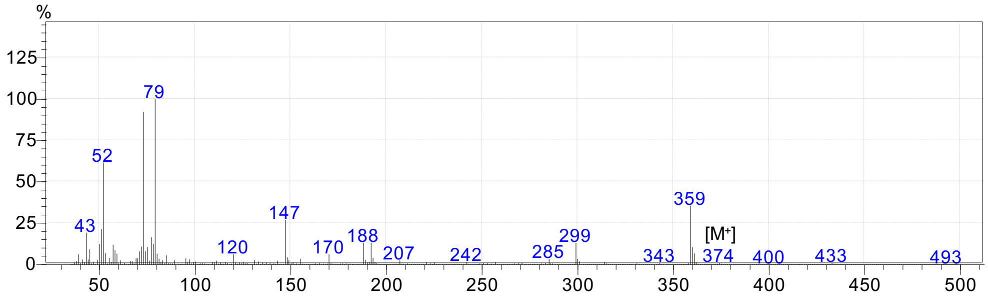

18) Tetradecane (Alk <u>C14</u>):

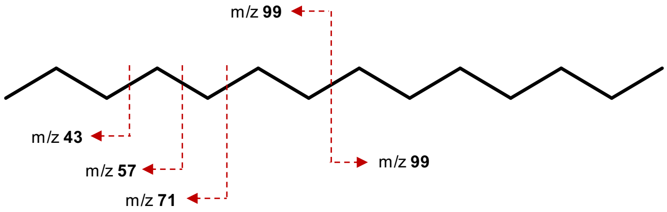

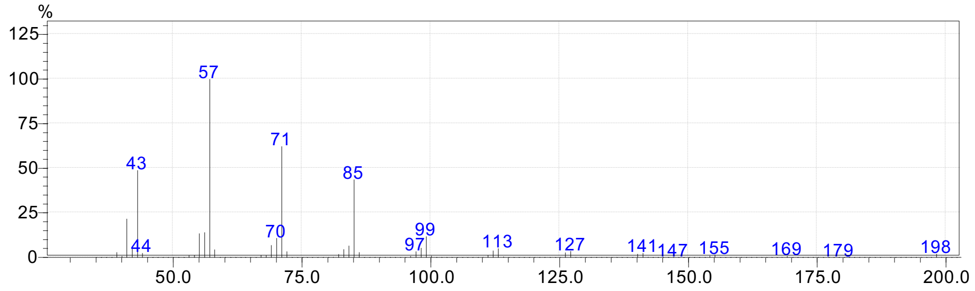

19) 2-keto (<u>C14</u>):

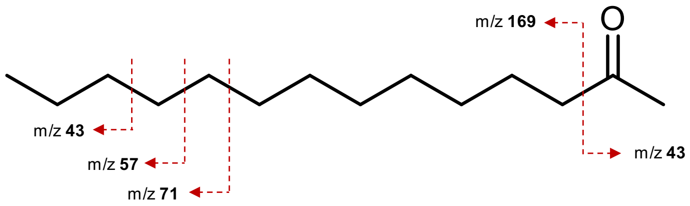

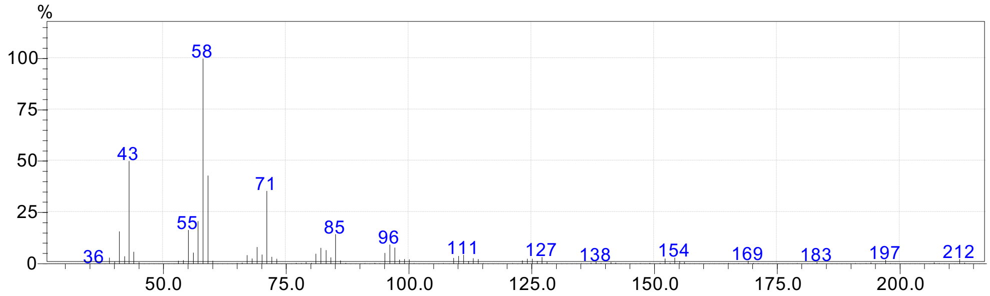

20) COOH (<u>C14</u>) (Myristic acid):

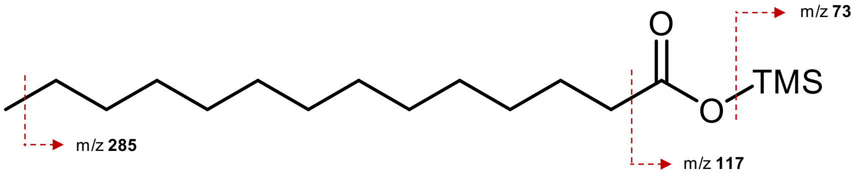

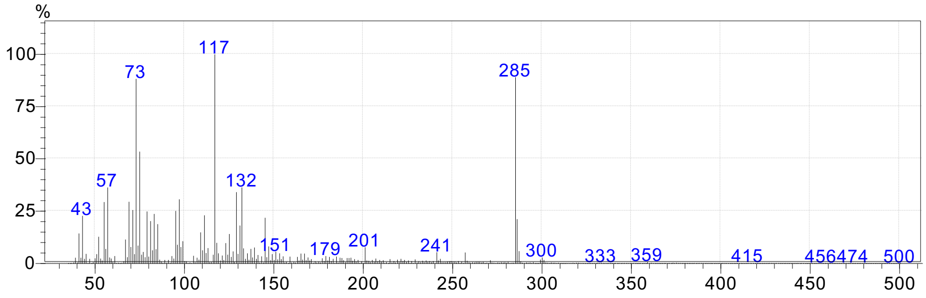

21) di-COOH (<u>C14</u>):

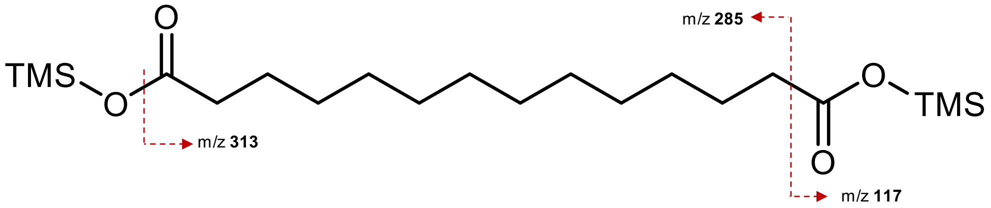

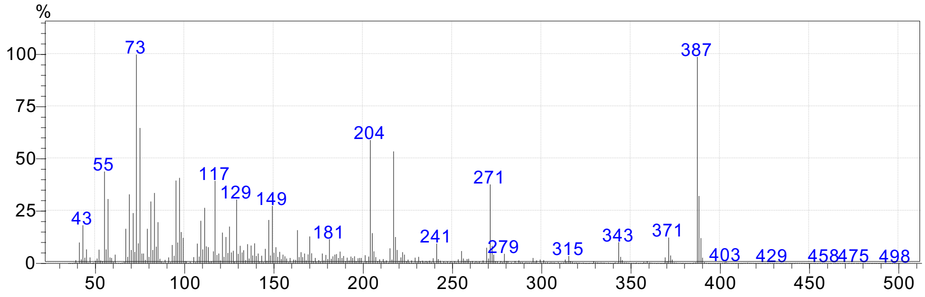

22) (ω-2)-keto-COOH (<u>C14</u>):

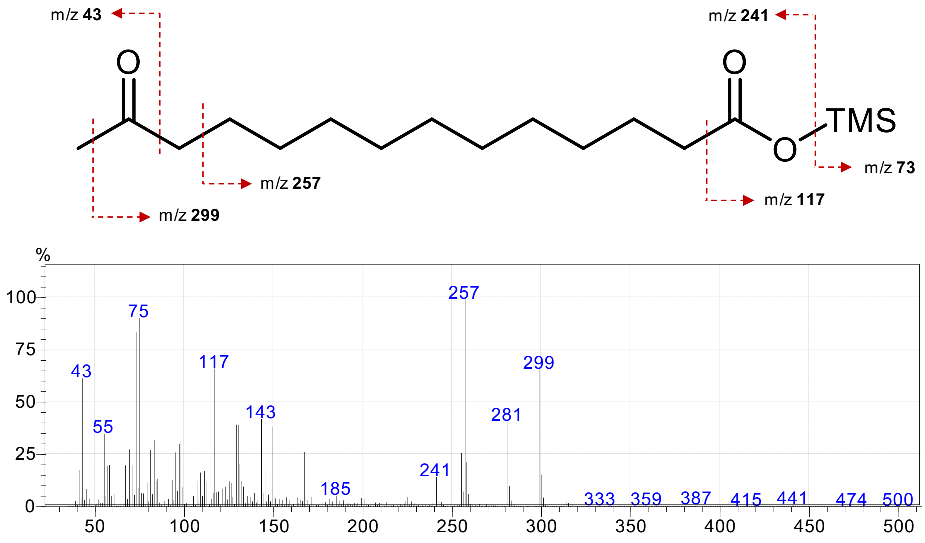

23) (ω)-OH-COOH (<u>C13</u>):

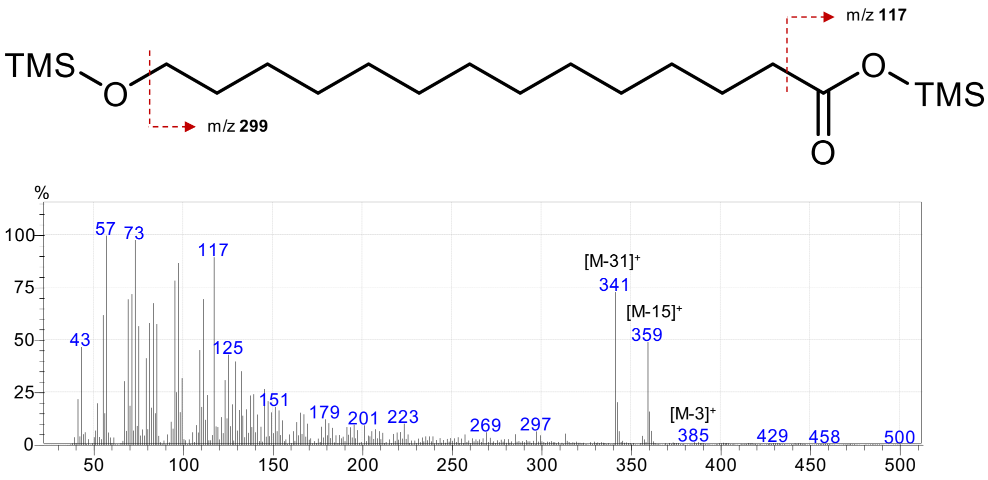

Supplement: Supplementary file 1 — cb4c00504_si_001.pdf [file cb4c00504_si_001.pdf]
